# The complex methylome of the human gastric pathogen *Helicobacter pylori*

**DOI:** 10.1093/nar/gkt1201

**Published:** 2013-12-02

**Authors:** Juliane Krebes, Richard D. Morgan, Boyke Bunk, Cathrin Spröer, Khai Luong, Raphael Parusel, Brian P. Anton, Christoph König, Christine Josenhans, Jörg Overmann, Richard J. Roberts, Jonas Korlach, Sebastian Suerbaum

**Affiliations:** ^1^Institute of Medical Microbiology and Hospital Epidemiology, Hannover Medical School, Carl-Neuberg-Straße 1, 30625 Hannover, Germany, ^2^German Center for Infection Research, Hannover-Braunschweig Site, Carl-Neuberg-Straße 1, 30625 Hannover, Germany, ^3^New England Biolabs, 240 County Road, Ipswich, MA 01938, USA, ^4^Leibniz Institute DSMZ-German Collection of Microorganisms and Cell Cultures, Inhoffenstraße 7B, 38124 Braunschweig, Germany and ^5^Pacific Biosciences, 1380 Willow Road, Menlo Park, CA 94025, USA

## Abstract

The genome of *Helicobacter pylori* is remarkable for its large number of restriction-modification (R-M) systems, and strain-specific diversity in R-M systems has been suggested to limit natural transformation, the major driving force of genetic diversification in *H. pylori*. We have determined the comprehensive methylomes of two *H. pylori* strains at single base resolution, using Single Molecule Real-Time (SMRT®) sequencing. For strains 26695 and J99-R3, 17 and 22 methylated sequence motifs were identified, respectively. For most motifs, almost all sites occurring in the genome were detected as methylated. Twelve novel methylation patterns corresponding to nine recognition sequences were detected (26695, 3; J99-R3, 6). Functional inactivation, correction of frameshifts as well as cloning and expression of candidate methyltransferases (MTases) permitted not only the functional characterization of multiple, yet undescribed, MTases, but also revealed novel features of both Type I and Type II R-M systems, including frameshift-mediated changes of sequence specificity and the interaction of one MTase with two alternative specificity subunits resulting in different methylation patterns. The methylomes of these well-characterized *H. pylori* strains will provide a valuable resource for future studies investigating the role of *H. pylori* R-M systems in limiting transformation as well as in gene regulation and host interaction.

## INTRODUCTION

The Gram-negative human pathogen, *Helicobacter pylori*, chronically infects more than half of the world population. *Helicobacter pylori* infection induces inflammation of the gastric mucosa, which can give rise to sequelae, such as peptic ulcer disease and gastric cancer (1). *Helicobacter pylori* is the bacterial pathogen with the highest genetic diversity and variability (2–4), which is believed to contribute to lifelong persistence by enabling adaptation to its host (2,3). In addition to a high mutation rate (5), recombination between different *H. pylori* strains during mixed infections with multiple strains within one stomach is the major driving force of allelic diversification (6–8). The naturally competent *H. pylori* differs from other bacteria by integrating unusually short fragments of DNA into its chromosome after natural transformation (9). The reasons for the small sizes of imports are largely unknown, but differences of the genomic content of active restriction-modification (R-M) systems have been suggested to limit recombination between *H. pylori* strains (10–12).

R-M systems are widely distributed among bacteria and are found in >90% of the analyzed genomes (13). Bacterial R-M systems were initially described as a defence mechanism against bacteriophage infection (14,15). They comprise two enzymatic activities: (i) a methyltransferase (MTase) activity that catalyzes the addition of a methyl group from the donor S-adenosyl methionine (SAM) to adenine or cytosine, and (ii) a restriction endonuclease (REase) activity that cleaves internal phosphodiester bonds of the DNA backbone. Both enzyme activities of the same system (cognate enzymes) recognize the same specific nucleotide sequence (recognition site), and methylation of the recognition site prevents restriction. The three major groups of R-M systems are classified as Type I, II and III, according to their subunit composition, cofactor requirements, structure of their recognition sequence and mode of action [for detailed reviews see (16,17)]. Type I systems are the most complex and form a heteropentamer (HsdR_2_M_2_S) that exerts three functions: restriction (HsdR), modification (HsdM) and specificity (HsdS). This complex works both as REase and MTase, but HsdM_2_S alone is sufficient for methylation. Sequence specificity of HsdR and HsdM is achieved by HsdS, which is typically composed of two target recognition domains (TRDs) mediating sequence recognition on both DNA strands. The simplest systems are the Type IIP R-M systems, which consist of two separate polypeptides (REase, MTase) that act independently of each other. Type III systems are also encoded by two genes (*mod* and *res*). While the Mod subunit alone achieves DNA modification, both subunits are required for restriction. In contrast to typical Type II MTases, which usually methylate 4–8 bp palindromic sites on both DNA strands, Mod catalyzes hemi-methylation of the DNA at 4–6 bp asymmetric recognition sites. More recently, a fourth class of R-M systems has been added. Type IV systems are encoded by one or two genes that represent methyl-dependent REases (18).

Adenine and cytosine are the only bases known to be enzymatically methylated. In bacteria, three types of methylation, *N*^6^-methyladenine (^m6^A), *N*^4^-methylcytosine (^m4^C) and 5-methylcytosine (^m5^C) have been detected. While Type I and Type III R-M systems only methylate adenine, all three types of methylation have been reported to be catalyzed by Type II MTases (17).

*Helicobacter pylori* genomes encode an unusually high number of R-M systems (13,19–21). The two first *H. pylori* strains whose genomes were sequenced are 26695 (19) and J99 (21), and their strongly different complements of R-M systems have been analyzed in some detail. The two strains have been proposed to encode members of all four types of R-M systems. While several studies have addressed the activity of Type II MTases (22–24), only one Type III MTase of *H. pylori* 26695 has been functionally characterized so far (25). Apart from that, Type I and Type III R-M systems of *H. pylori* were mostly uncharacterized and their specificity unknown. An overview about known and predicted R-M genes for many *H. pylori* strains can be found in the REBASE database [http://rebase.neb.com/rebase/rebase.html, (13)].

It has recently been reported that DNA methylation can be reliably detected at single-base resolution by Single Molecule Real-Time (SMRT®) sequencing technology, which enables the genome-wide detection of ^m6^A, ^m4^C and ^m5^C methylation (26,27). In this next-generation sequencing technology, genome sequencing is achieved by monitoring the action of an engineered phi29-based DNA polymerase, which catalyzes the incorporation of fluorescently labeled nucleotides. Besides the primary sequence, alterations in the kinetics of the polymerase allow the identification of methylated nucleotides in the template DNA (26). This method has recently been used to characterize MTase activity on the whole-genome level (28–30). Here, we have used SMRT sequencing to characterize the complete methylomes of the *H. pylori* strains 26695 and J99-R3 [rifampicin-resistant mutant of strain J99, (31)] and have identified novel features of MTases encoded by these strains.

## MATERIALS AND METHODS

### Bacterial strains and culture conditions

All *Escherichia coli* and *Helicobacter pylori* strains used in this study are listed in Supplementary Table S1. *Helicobacter pylori* strains were cultured on blood agar plates with antibiotic supplements as previously described (32). Strain J99-R3 was chosen for this study rather than J99 so that the MTase mutants generated in the J99-R3 background can later be used in functional studies about the impact of DNA methylation on transformation where the rifampicin resistance serves as a selectable marker.

*Escherichia coli* strains were cultured in LB broth or on LB plates (Lennox L Broth, Invitrogen™—Life Technologies, Darmstadt, Germany) supplemented with ampicillin (200 µg/ml) and/or kanamycin (20 µg/ml) as required.

### DNA techniques

All standard procedures (cloning, DNA amplification, purification and manipulation) were performed according to standard protocols (33). Large-scale purification of chromosomal DNA from *H. pylori* was performed using QIAGEN Genomic-tip 100/G columns (QIAGEN, Hilden, Germany). Plasmid DNA from *E. coli* strains was isolated using QIAGEN tip 100 columns, and chromosomal DNA from *E. coli* strains was purified using phenol/methylene chloride extraction.

### SMRT® sequencing

SMRTbell™ template libraries were prepared according to the instructions from Pacific Biosciences, Menlo Park, CA, USA, following the Procedure & Checklist for 10 kb Template Preparation and Sequencing and Procedure & Checklist for 1 kb Template Preparation and Sequencing, respectively. For 10-kb libraries, one 90-min movie was taken, for 800-bp libraries two 45-min movies.

Detection of ^m5^C signals by SMRT sequencing requires pretreatment of fragmented DNA (800 bp) to enhance the signature. Conversion of ^m5^C to 5-carboxycytosine (^5ca^C) before sequencing was achieved by treatment of samples with ten-eleven translocation (TET) proteins (34) using the Tet1 Oxidation kit (Wisegene, Chicago, IL, USA). Sheared DNA (500 ng) was Tet1 treated following the instructions of the supplier. Purified DNA was used for SMRTbell preparation starting with end repair.

### Bioinformatic analyses of SMRT® sequencing data

Genome-wide detection of base modification and the affected motifs was performed using the standard settings in the ‘RS_Modification_and_Motif_Analysis.1’ protocol included in SMRT Portal version 1.3.3, 1.4 and 2.0.1. Within those protocols FASTA exports of GenBank/RefSeq entries NC_000915.1 and NC_000921.1 were used as references for *H. pylori* 26695 and J99, respectively. While ^5ca^C yields a strong kinetic signature in SRMT sequencing, for reasons beyond the scope of this article, the TET conversion is not 100% at every genomic location and appears to be sequence context dependent. Hence, it precludes using the same kinetic score threshold as for the ^m6^A detection to call a site as modified. For the parental strains and TET-treated samples, a kinetic score threshold of Quality value (QV) 100 and 50, respectively, was used such that the mean false discovery rate (FDR) is ∼1% across all four canonical bases. For the mutants, a threshold of QV 40 was used.

Strains *H. pylori* 26695 and J99-R3 were sequenced on four SMRT Cells yielding a coverage of 350×. For detection of ^m5^C, three SMRT Cells were sequenced leading to 180× coverage for each of both strains. ^m5^C modifications were detected by comparative sequencing of TET-treated samples and untreated ones using the latter as ‘control jobs’ in SMRT Portal. Modification.gff files were used as input for Motif Finder software. The knockout mutants generated in this study were run on single SMRT Cells with ∼80× coverage. While the high sequencing coverage ensures proper statistics for every genomic position for genome-wide profiling, the lower coverage used for the mutants was sufficient for the binary determination of MTase activity (‘on’ versus ‘off’) in the mutant. In SMRT Portal, each of these mutants was compared (i) to the *in silico* model to get a list of all affected motifs and (ii) to the corresponding parental strains to capture the effect of gene inactivation on the methylome. In the latter case, parental strains were reanalyzed using the Job IDs of the corresponding mutant as ‘Control Job ID’. To confirm the presence or absence of motifs, modifications.gff and/or raw modifications.csv files obtained through ‘RS_Modification_and_Motif_Analysis.1’ protocol were screened using the windows version of the MotifMaker program (https://github.com/PacificBiosciences/MotifMaker) as well as publicly available R-scripts (https://github.com/PacificBiosciences/motif-finding).

### Insertion mutagenesis in *H. pylori*

Mutant construction by natural transformation-mediated allelic exchange was performed as described previously (35–37). Oligonucleotides used for mutagenesis are provided in Supplementary Table S2. Briefly, the target genes were amplified by polymerase chain reaction (PCR) and ligated to pUC19. The resulting plasmids were used as templates for inverse PCR reactions, designed to delete a central part of the target gene. Finally, a kanamycin resistance cassette [*aphA-3* (38)] was introduced (Supplementary Table S4). The direction of transcription of the *aphA-3* resistance gene was the same as that of the target gene to avoid possible polar effects (39). Plasmids containing the interrupted gene were used as suicide plasmids for natural transformations of the *H. pylori* strains 26695 and J99-R3. The successful chromosomal replacement of the target gene with the disrupted gene construct via allelic exchange (double crossover) was checked by PCR using suitable primer combinations.

### Overexpression of candidate genes in *E. coli*

Selected MTase and specificity subunit (*hsdS*) genes were amplified via PCR using Q5® Hot Start High-Fidelity Polymerase (NEB, Ipswich, MA, USA). Oligonucleotides are provided in Supplementary Table S3. PCR products were ligated to PCR-amplified pRRS or pACYC184 vectors using the Gibson Assembly™ technique (NEB, Ipswich, MA, USA) described previously (28). The putative MTase JHP1409 was amplified via PCR using Phusion DNA polymerase (NEB, Ipswich, MA, USA) and ligated to pRRS. The resulting constructs (Supplementary Table S4) were overexpressed in the nonmethylating host *E. coli* ER2796 (*dam^−^ dcm^−^*) or in *E. coli* ER2683 (*dam*^+^ *dcm*^+^) (NEB, Ipswich, MA, USA). Expression of the candidate genes was achieved by the lac promoter present on pRRS and by the tet promoter present on pACYC184.

Site-specific mutagenesis of the relevant expression plasmids was performed to correct frameshift mutations present in some of the MTase genes. For this, frameshifts were corrected via inverse PCR. Nucleotides were inserted or deleted within the repeat, and additional synonymous polymorphisms were introduced to stabilize the repeats (e.g. CCC CCC CCC was changed to CCA CCT CCT). Sequence alterations were confirmed by targeted Sanger sequencing. All candidate wild-type proteins and enzymes obtained by frameshift repair were pretested for activity in a methyltransferase activity assay. If a cloned MTase product showed the ability to incorporate ^3^H-methyl groups into DNA using tritiated SAM as substrate, the genomic DNA (gDNA) of the *E. coli* host was subjected to SMRT sequencing to determine the resulting methylation pattern.

### Endonuclease and methyltransferase activity assay

*Escherichia coli* clones expressing MTase and/or REase genes were grown, harvested and disrupted by sonication for crude extract preparation. The crude extracts were tested for endonuclease (40) and/or methyltransferase (41) activity as described previously.

### Purification of JHP1272 endonuclease

A crude extract of *E. coli* ER2683 hosting an expression plasmid carrying the fully repaired JHP1272 gene (pSUS3093) was prepared from 2 l of culture as described above, and passed through a 5 ml HiTrap™ Heparin HP column (GE Healthcare, Piscataway, NJ, USA) for purification as described previously (42). Column fractions containing the enzyme were combined, diluted to 50 mM NaCl and further purified over a 6 ml Resource S column (GE Healthcare, Piscataway, NJ, USA). The Resource S column fractions containing JHP1272-mut1 were dialyzed against storage buffer (50 mM KCl, 10 mM Tris–HCl, 1 mM DTT, 0.1 mM EDTA, 50% glycerol (pH 7.4 at 25°C) and used for subsequent analysis.

## RESULTS

### Methylome analysis of *H. pylori* 26695 and J99-R3 by SMRT® sequence analysis

**Figure 1.**
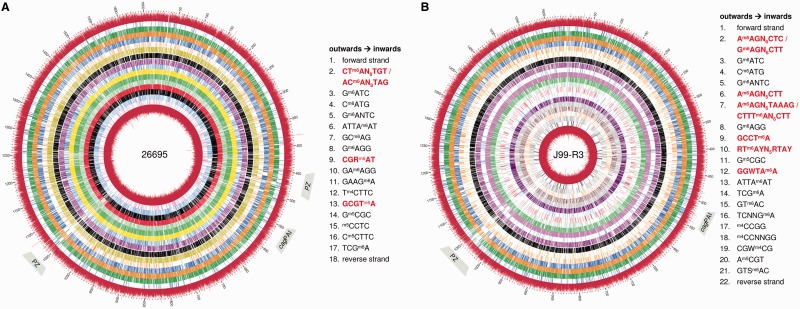
Circos plots displaying the distribution of methylated bases in the genomes of *H. pylori* 26695 (**A**) and J99-R3 (**B**). The outermost and innermost tracks represent the interpulse duration ratios. The colored tracks in between represent the location of methylation of the different motifs. Each motif is displayed by one track except for the asymmetric Type I recognition sites for which both motifs were combined. For ^m6^A and ^m4^C methylation, the cutoff for calling a site modified was Qmod >100 (kinetic score); for the TET-treated ^m5^C methylation a kinetic score threshold >50 was used. The position and type of methylation for each motif is depicted in the legend. Novel motifs are highlighted in red. The genome tick marks display the genomic positions (kbp). The locations of the *cag* pathogenicity island (cagPAI) and PZ are indicated. Please note that the PZ of strain 26695 is split into two regions (21).

The distribution of methylated bases in the genomes of *H. pylori* strains 26695 and J99-R3, a rifampicin-resistant mutant of strain J99 (31) was analyzed using SMRT® sequencing technology. ^m6^A and ^m4^C modifications can be detected with high accuracy by analysis of the raw data generated in the process of SMRT sequencing without additional sample treatment. Genomic positions 50 533 and 57 345 were found to be methylated (either ^m6^A or ^m4^C) with high probability (kinetic score >100) in 26695 and J99-R3, respectively (Figure 1 and Supplementary Figure S1). The majority of these methylations were ^m6^A modifications (26695: ^m6^A—92.1%, ^m4^C—7.9%; J99-R3: ^m6^A—89.1%, ^m4^C—10.9%). Both *H. pylori* strains also encode ^m5^C MTases. Detection of ^m5^C signals was achieved by Tet1 mediated conversion of ^m5^C to ^5ca^C. However, the fraction of ^m5^C methylation observed was substantially lower than those for ^m4^C and ^m6^A motifs, but this is likely to be due to technical reasons (see ‘Materials and Methods’ section).

The sequence and methylation data were analyzed for methylation patterns with Pacific Biosciences’ Motif Finder software. In the case of ^m6^A and ^m4^C modifications, Motif Finder analysis also provides information about the position of the modified nucleotide within the recognition site. In total, 32 unique methylated motifs were identified in the two strains (7 shared motifs, 10 26695-specific motifs, 15 J99-R3-specific motifs) for all three types of methylation (Figure 1). Recognition sites of R-M systems can comprise one motif for palindromic sites or two motifs for nonpalindromes, and we refer to recognition sites rather than motifs throughout the article and in the tables to facilitate reading.

Thirteen distinct recognition sequences (corresponding to 17 methylated motifs) were identified in *H. pylori* 26695 (Figure 1A). Ten of the recognition sites matched known Type II and III ^m4^C, ^m5^C and ^m6^A MTase activities (Table 1). However, three of them could not be assigned to any previously known or predicted 26695 MTase specificity [GCGT^m6^A, CGR^m6^AT, AC^m6^AN_8_TAG; the methylated base is indicated (e.g. ^m6^A) and, if present, an underlined nucleotide indicates methylation of the position on the complementary strand] (Figure 1A and Table 1).

**Table 1.** Methylated sequence motifs detected for *H. pylori* 26695

Type of R-M system	Methyltransferase activity^a^	Type of methylation	Total number in genome^b^	Number methylated	% detected methylated	Assignment	Locus	Reference
I	5′-AC**A**N_8_TAG-3′*	^m6^A	330	326	98.8	M.HpyAXIII^c^	hp0850	This study
	3′-TGTN_8_**A**TC-5′*		330	326	98.8			
II	5′-**C**CTC-3′	^m5^C	4885	3913	80.1	M2.HpyAVI	hp0051	(23)/(24)
	3′-GG**A**G-5′	^m6^A	4885	4841	99.1	M1.HpyAVI	hp0050	(23)
	5′-C**C**TTC-3′	^m5^C	1304	1198	91.9	M.HpyAV	hp0054	This study/(23)
	3′-GG**A**AG-5′	^m6^A	1304	1252	96.0			
	5′-G**A**TC-3′	^m6^A	10 834	10 764	99.4	M.HpyAIII	hp0092	(23)
	5′-TCG**A**-3′	^m6^A	608	601	98.8	M.HpyAX	hp0260	(24)
	5′-ATTA**A**T-3′	^m6^A	972	957	98.5	M.HpyAVII	hp0478	(23)
	5′-G**C**GC-3′	^m5^C	12 538	6328	50.5	M.HpyAVIII	hp1121	(23,24)
	5′-C**A**TG-3′	^m6^A	14 782	14 741	99.7	M.HpyAI	hp1208	(23)
	5′-G**A**NTC-3′	^m6^A	5570	5549	99.6	M.HpyAIV	hp1352	(23)
	5′-GAAG**A**-3′	^m6^A	4823	4809	99.7	M1.HpyAII	hp1367	(23)
	3′-CTT**C**T-5′	^m4^C	4823	4593	95.2	M2.HpyAII	hp1368	
	5′-GCGT**A**-3′*	^m6^A	2058	2046	99.4	HpyAXIV	hp1517	This study
III	5′-GC**A**G-3′	^m6^A	4260	4234	99.4	M.HpyAXI	hp0593	(25)
IIG or III	5′-CGR**A**T-3′*	^m6^A	3275	3240	98.9	-	n.d.	This study

^a^The methylated position within the motif is highlighted in bold. Underlining indicates the modified base in the complementary strand. Pairs of reverse-complementary motifs belonging to one recognition sequence are grouped together. Novel recognition sequences are highlighted by an asterisk.

^b^The total number includes motifs occurring on the ‘+’- and ‘−’-strand.

^c^The MTase M.HpyAXIII achieves specificity from distantly located S subunit S.HpyAXIII (HP0790).

For strain J99-R3, 20 recognition sequences (22 motifs) were detected (Figure 1B). Fourteen recognition sites matched known MTase activities, while six were unknown [A^m6^AGN_6_CTC, A^m6^AGN_6_TAAAG, A^m6^AGN_5_CTT, GCCT^m6^A, GGWT^m6^AA and RT^m6^AYN_5_RTAY] (Figure 1B and Table 2).

**Table 2.** Methylated sequence motifs detected for *H. pylori* J99-R3

Type of R-M system	Methyltransferase activity^a^	Type of methylation	Total number in genome^b^	Number methylated	% detected methylated	Assignment	Locus	Reference
I	5′-A**A**GN_6_CTC-3′*	^m6^A	611	610	99.8	M.Hpy99XVII^c^	jhp0786	This study
	3′-TTCN_6_G**A**G-5′*		611	610	98.8			
	5′-RT**A**YN_5_RTAY-3′*	^m6^A	370	363	98.1	M.Hpy99XVI^c^	jhp0786	This study
	5′-A**A**GN_5_CTT-3′*	^m6^A	1582	1575	99.6	M.Hpy99XV^d^	jhp1423	This study
	5′-A**A**GN_6_TAAAG-3′*	^m6^A	287	285	99.3	M.Hpy99XV^d^	jhp1423	This study
	3′-TTCN_6_**A**TTTC-5′*		287	286	99.7			
II	5′-CTCC-3′	^m6^A	5027	4993	99.3	M1.Hpy99V^e^	jhp0043	(22)
	5′-GTS**A**C-3′	^m6^A	210	203	96.7	M.Hpy99II	jhp0045	(22)
	5′-G**A**TC-3′	^m6^A	10 958	10 934	99.8	M.Hpy99VI	jhp0085	(22)
	5′-TCG**A**-3′	^m6^A	674	653	96.9	M.Hpy99VII	jhp0244	(22)
	5′-**C**CGG-3′	^m4^C	3624	3581	98.8	M.Hpy99VIII	jhp0248	(22)
	5′-ATTA**A**T-3′	^m6^A	854	841	98.5	M.Hpy99XIX	jhp0430	(22)/This study
	5′-A**C**GT-3′	^m5^C	1000	765	76.5	M.Hpy99XI	jhp0435	(22)
	5′-GT**A**C-3′	^m6^A	368	355	96.5	M.Hpy99XII	jhp0454	(43)
	5′-**C**CNNGG-3′	^m4^C	2398	2323	96.9	M.Hpy99IV	jhp0629	(22)
	5′-CGW**C**G-3′	^m4^C	538	454	84.4	M.Hpy99I	jhp0756	(22)
	5′-TCNNG**A**-3′	^m6^A	3906	3838	98.3	M.Hpy99XVIII	jhp1012	(22)/This study
	5′-G**C**GC-3′	^m5^C	13 798	6917	54.0	M.Hpy99III	jhp1050	(22)/This study
	5′-C**A**TG-3′	^m6^A	15 120	15 091	99.8	M.Hpy99X	jhp1131	(22)
	5′-G**A**NTC-3′	^m6^A	5516	5490	99.5	M.Hpy99IX	jhp1271	(22)
	5′-GGWTA**A**-3′*	^m6^A	2676	2655	99.2	Hpy99XIV	jhp1272	This study
	5′-GCCT**A**-3′*	^m6^A	3106	3096	99.7	Hpy99XIII	jhp1409	This study

^a^The methylated position within the motif is highlighted in bold. Underlining indicates the modified base in the complementary strand. Pairs of reverse-complementary motifs belonging to one recognition sequence are grouped together. Novel recognition sequences are highlighted by an asterisk.

^b^The total number includes motifs occurring on the ‘+’- and ‘−’-strand.

^c^JHP0786 (M) can form two distinct Type I MTase complexes and exhibits a dual specificity (methylation of two recognition sites) depending on the associated S subunit. The MTase complex composed of JHP0786 and JHP0785 (S) was designated M.Hpy99XVI and the one containing JHP0786 and JHP0726 (S) was named M.Hpy99XVII.

^d^This Type I MTase complex has dual specificities caused by the presence of three TRDs in the associated S subunit (JHP1422).

^e^For Hpy99V, there are two MTases associated. M1.Hpy99V is an active ^m6^A MTase methylating GGAG while M2.Hpy99V is an inactive ^m5^C MTase as the TET-treated samples showed no methylation of its recognition site CTCC.

The Motif Finder analysis also provided information about the fraction of motifs that are methylated. Overall, most of the target sequences were almost fully methylated (>98%, Tables 1 and 2 and Supplementary Table S7). As noted above, the TET conversion from ^m5^C to ^5ca^C is not 100%, preventing a reliable quantification of the methylation rate of ^m5^C motifs with this method (see ‘Materials and Methods’ section).

### Comparison of *H. pylori* methylome analyses with previous findings

The majority of identified recognition sites was in agreement with previous reports of MTase activities of Type II R-M systems in *H. pylori* (22–24). Where discrepancies were noted, we performed functional studies with the aim to resolve these. HP0910 (M.HpyAIX, GTNNAC) was previously reported to be active based on overexpression in *E. coli* (23). In our study, HP0910 was not active since methylation of the GTNNAC site was not detected in strain 26695. Sequence analysis revealed no differences compared with the published 26695 HP0910 gene and its flanking regions. Restriction analysis with Hpy166II, an isoschizomer of the cognate REase HP0909, showed complete digestion of 26695 gDNA (Supplementary Figure S2A), consistent with a lack of methylation of GTNNAC. To further elucidate the activity of HP0910, the gene was overexpressed in *E. coli* in two different systems. Expression of HP0910 in pADC containing the strong *ureA* promoter did not confer resistance to REase cleavage (Supplementary Figure S2B). Furthermore, expression of HP0910 and of HP0909-HP0910 in pRRS did neither lead to ^3^H-SAM incorporation into DNA nor to observation of endonuclease activity for the joint construct of REase and MTase. Based on our results, we conclude that HP0910 is not active in 26695.

In J99-R3, we detected methylation of the proposed recognition sites of the predicted MTases JHP0430 and JHP1012 (ATTAAT and TCNNGA, respectively), which were previously reported to be inactive (22). To elucidate whether the two gene products are active MTases in J99-R3, knockout experiments were performed. We individually inactivated both open reading frames (ORFs) in J99-R3, prepared gDNA and performed digestion experiments with isoschizomers of the cognate REases. The gDNA of both mutant strains was completely digested after incubation with either AseI (isoschizomer of JHP0431, cleaves ATTAAT) or Hpy188III (JHP1013, cleaves TCNNGA) (Supplementary Figure S2C), confirming the presence of active JHP0430 and JHP1012 in J99-R3. We named these newly characterized MTase activities M.Hpy99XIX (JHP0430) and M.Hpy99XVIII (JHP1012).

### Characterization of novel MTase specificities

To identify the MTases that recognize and methylate the nine novel recognition sequences (3 in 26695 and 6 in J99-R3), 13 MTase candidate genes (plus six genes encoding candidate S subunits) were selected using the data contained in the REBASE database [http://rebase.neb.com/rebase/rebase.html, (13)]. Candidate genes were disrupted via introduction of a kanamycin resistance cassette, and gDNA from the mutant strains was subjected to SMRT sequencing so that their methylation patterns could be compared with those of the parental strains. As proof of principle, we inactivated two known Type II MTases of J99-R3 (JHP0085—GATC, JHP1271—GANTC), which resulted in the expected complete loss of methylation at the target sequences (Supplementary Figure S3). In addition to functional inactivation, we overexpressed several candidate genes in suitable *E. coli* strains and tested their activity by an MTase assay and/or SMRT sequencing of either the expression plasmid or the gDNA of the expression strain.

Using this combined approach, two out of three novel recognition sequences in 26695 and all six novel sites in J99-R3 could be assigned to previously uncharacterized MTases. In the following paragraphs, we summarize the results for each system for which new information was obtained, starting with 26695 systems, followed by systems identified in J99-R3.

#### HP0850/HP0790

Inactivation of the putative Type I MTase gene HP0850 resulted in the loss of ACAN_8_TAG methylation (Supplementary Table S5). Type I MTases require association with a specificity subunit (S subunit) for sequence recognition. HP0850 is located next to two ORFs with homology to adjacent parts of an S subunit (HP0848-HP0849). However, a genuine frameshift (confirmed in 26695) splits the sequence into two ORFs, which should prevent its translation. Thus, we hypothesized that HP0850 might associate with an alternative S subunit. Functional inactivation of HP0848-HP0849 and the two distantly located putative S subunits HP0462 and HP0790 revealed that HP0790 targets sequence specificity to HP0850 (Supplementary Table S5). This finding demonstrates that a Type I MTase can partner with an S subunit that is not encoded adjacently on the chromosome. We named this newly characterized MTase M.HpyAXIII (Table 1) and its adjacent inactive S subunit S.HpyAXIIIN (N for Non-functional). The associated active S subunit HP0790 was designated S.HpyAXIII.

#### HP1517

Functional inactivation of HP1517 (Type IIG MTase) resulted in the loss of GCGTA methylation (Supplementary Table S5). Similar to other Type IIG MTases, only hemi-methylation of the recognition site was detected. This MTase has 67% amino acid identity with JHP1409 of J99-R3. HP1517 was named HpyAXIV (Table 1).

#### JHP0414/JHP0415

**Figure 2.**
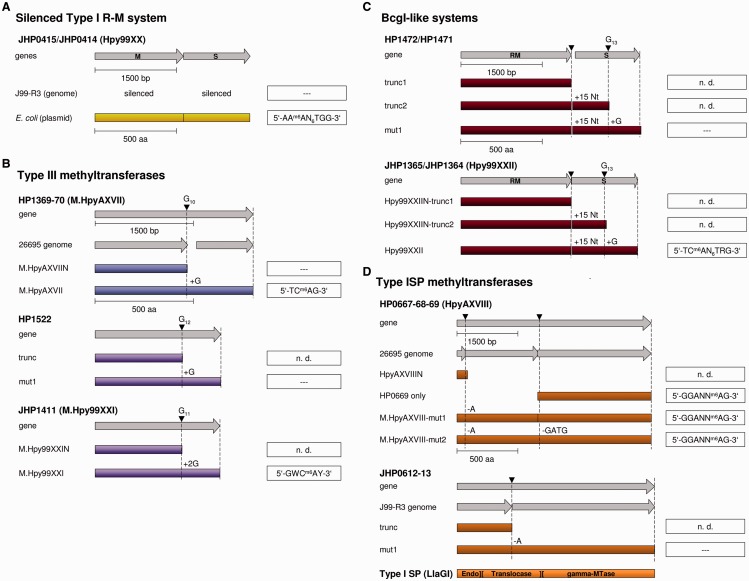
Graphical representation of the silenced JHP0414/JHP0415 Type I MTase of J99-R3 (**A**), the putative phase-variable Type III MTases (**B**), BcgI-like R-M systems (**C**) and Type ISP MTases (**D**). (A) The gene sequences of HsdM (JHP0415) and HsdS (JHP0414) are displayed as gray bars and the protein sequences as yellow bars. SMRT sequencing of the J99-R3 genome did not reveal methylation by the Type I MTase complex (dashed lines), while expression of the enzyme complex on a plasmid in *E. coli* 2796 and subsequent SMRT analyses demonstrate methylation of the recognition site AAAN_6_TGG. (B–D) The gene sequences (gray arrows) contain one or two putative or authentic frameshifts that would prevent translation of full-length proteins (colored bars). Each frameshift was repaired through site-directed mutagenesis (addition and deletion of an indicated number of nucleotides, +15 Nt—TAAGGTTAATATATG) and correction was verified by targeted Sanger sequencing. The activity of the MTase proteins was pretested with a methylation activity assay. If an MTase showed activity in the assay, the gDNA of the corresponding *E. coli* host strain ER2796 was subjected to SMRT sequencing and analyzed for methylation. ‘mut’ marks alleles with frameshift corrections, ‘trunc’ labels not fully translated alleles, ‘n.d.’ points out that no SMRT sequencing was performed and dashed lines indicate that no methylation could be detected through SMRT sequencing. Filled inverted triangles highlight the position of the frameshift mutations. JHP—ORFs of J99, HP—ORFs of 26695. Homologous systems are displayed in the same color.

To investigate the function of this yet uncharacterized putative Type I R-M system of J99-R3, we disrupted the S subunit JHP0414. Functional inactivation did not result in a change of the methylation pattern (Supplementary Table S6), although the JHP0414 ORF contains no mutations compared with the published sequence and should enable translation of a full-length protein. Overexpression of JHP0414 (HsdS) and JHP0415 (HsdM) in *E. coli* followed by SMRT sequencing of gDNA of the expression strain revealed methylation of the recognition site AA^m6^AN_6_TGG by JHP0414/JHP0415 (Figure 2A), which, however, was not detected in the methylome of J99-R3. Thus, this MTase is functional, but silenced in the *H. pylori* J99-R3 genome, perhaps because the frameshift in the endonuclease gene upstream of the MTase and S subunit disrupts the transcription or translation of these otherwise intact genes. This is the first such example of silencing of a Type I system. This R-M system was named Hpy99XX.

#### JHP0785/JHP0786/JHP0726

**Figure 3.**
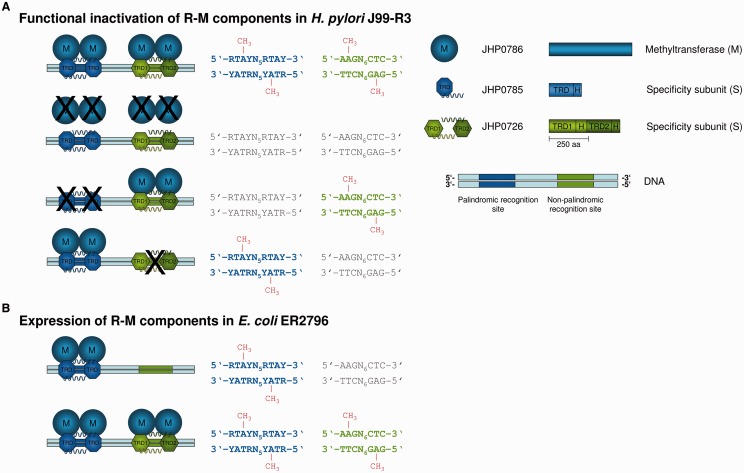
Functional characterization of JHP0785/JHP0786 and JHP0726 through insertion mutagenesis (**A**) and expression of the R-M genes in *E. coli* 2796 (**B**) confirmed that the Type I MTase JHP0786 interacts with the two S subunits JHP0785 or JHP0726 to achieve methylation of two different recognition sites. The methylation patterns of the J99-R3 parental and mutant genomes and the *E. coli* ER2796 host genomes were analyzed by SMRT sequencing. Motifs displayed in gray are not modified. Methylation of a recognition site is indicated by coloring and boldface type. The methylated nucleotide within a site is highlighted by the addition of a methyl group (CH_3_) to the sequence. ‘M’ is the abbreviation for methyltransferase, which acts as a homodimer. The two S subunits are composed of either one or two TRDs and interaction of TRDs is achieved by helical connector regions (H) adjacent to each TRD. (A) Expression of the three R-M components in J99-R3 and functional inactivation of either component (labeled by a black cross) resulted in different methylation patterns of the two distinct recognition sites. (B) Analysis of enzyme activity through plasmid-based expression of either JHP0786/JHP0785 (pRRS) alone or together with JHP0726 (pACYC184) in *E. coli*.

A strain carrying a disruption in the Type I MTase JHP0786 (HsdM) lost the ability to methylate two distinct recognition sequences (AAGN_6_CTC and RTAYN_5_RTAY) (Figure 3A and Supplementary Table S6). The adjacent HsdS protein JHP0785 contains only one TRD, which could enable recognition of a palindromic sequence like RTAYN_5_RTAY (Figure 3), but was extremely unlikely to mediate recognition of a further nonpalindromic site. Functional inactivation of JHP0785 confirmed this assumption (Figure 3A and Supplementary Table S6). We hypothesized that a different S gene might be responsible for the recognition of the nonpalindromic sequence AAGN_6_CTC. We inactivated the orphan S gene JHP0726 (no HsdR and HsdM subunit associated), which resulted in a loss of AAGN_6_CTC methylation, but did not affect RTAYN_5_RTAY (Figure 3A). To confirm this result, we overexpressed JHP0785/JHP0786 (HsdS/HsdM) in pRRS and JHP0726 (HsdS) in the compatible vector pACYC184 and performed interaction studies in *E. coli*. Overexpression of the three R-M subunits and subsequent SMRT sequencing of the host gDNA revealed methylation of both recognition sequences, while overexpression of JHP0785/JHP0786 (S/M) alone led to the detection of RTAYN_5_RTAY methylation (Figure 3B). This is, to our knowledge, the first time that an interaction of two different S genes, encoded remotely on the genome, with one M gene could be shown. Due to the novelty of this finding, we propose an adapted nomenclature to distinguish between the two MTase activities of JHP0786 depending on its association with the two different S-subunits. The MTase composed of JHP0786 and JHP0785 was designated M.Hpy99XVI and the one containing JHP0786 and JHP0726 was named M.Hpy99XVII.

#### JHP1422/JHP1423

**Figure 4.**
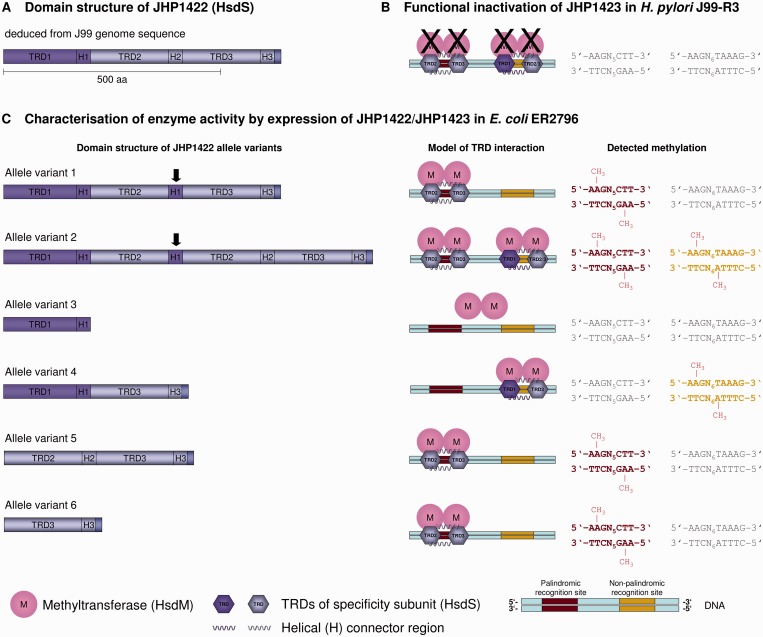
Graphical representation of the unique ability of the Type I MTase complex JHP1422/JHP1423 (HsdS/HsdM) to recognize and methylate two distinct recognition sites. (**A**) Schematic representation of the domain structure of the JHP1422 specificity subunit (HsdS) deduced from the J99 genome sequence (21). The proposed protein harbors three TRDs of which TRD1 is unique and TRD2 and 3 are duplicated as indicated by the coloring. Helical connector regions (H), known as conserved regions (CR), flank each TRD. (**B**) Inactivation of JHP1423 (HsdM) by insertion mutagenesis and subsequent SMRT analysis resulted in a lack of methylation of two different recognition sites as indicated by the gray color of the two sites. (**C**) Joint expression of JHP1423 (M) and several allele variants of JHP1422 led to different methylation patterns in the *E. coli* host genome. Cloning of JHP1422/JHP1423 yielded two naturally occurring allelic variants of JHP1422 (left panel: allele variant 1 and 2, JHP1423 sequence not shown) that differ in composition and protein length. In contrast to the proposed sequence, the H2 region following TRD2 is replaced by the H1 for allele variant 1. Allele variant 2 shows the same exchange but, in addition, has a second TRD2 flanked by the H2 region. Both variants have a different impact on methylation of the two recognition sites (right panel). Allele variants 3–6 were obtained by mutagenesis to reveal the specificity of the TRDs. Coloring and boldface type highlight methylation of the recognition sites, and the methyl group (CH_3_) indicates the modified nucleotide. Recognition sequences displayed in gray are not modified. The methylation patterns were analyzed by SMRT sequencing of the J99-R3*jhp1423* mutant genome (B) and *E. coli* ER2796 genomes (C).

Surprisingly, mutation in another Type I MTase, JHP1423, also led to the abrogation of methylation of two distinct recognition sites (AAGN_5_CTT and AAGN_6_TAAAG) (Figure 4B and Supplementary Table S6). To confirm these results, we overexpressed the MTase and its adjacent S subunit (JHP122) in *E. coli* ER2796. SMRT sequencing showed methylation of both recognition sites in the *E. coli* host genome (Figure 4C). The unique ability of this Type I R-M system to recognize and modify two different recognition sites can be explained by the unusual presence of three consecutive TRDs within the JHP1422 S protein (Figure 4A). While the first TRD is unique, the latter two are identical. During the cloning of PCR-amplified JHP1422/JHP1423 we obtained two different allelic variants of the JHP1422 sequence (Figure 4C). Expression of these variants resulted in different phenotypes. While allele variant 1 catalyzed methylation of the palindromic site only, expression of allele variant 2 led to modification of both recognition sites (Figure 4C). We hypothesized that by combining two of the three TRDs, different recognition sites might be targeted and modified. We therefore created S subunit constructs containing different TRD combinations and expressed these together with the JHP1423 MTase in *E. coli* (Figure 4C, allele variants 3–6). TRD1 and TRD3 together (allele variant 4) modified the nonpalindromic AAGN_6_TAAAG site, while TRD2 and TRD3 together (allele variant 5), and TRD3 alone (allele variant 6), both modified the palindromic AAGN_5_CTT site. Thus, TRD1 is responsible for recognition of the CTTT^m6^A half site, while the identical TRD2/TRD3 recognizes the A^m6^AG half site. TRD3 alone was able to recognize the palindromic site (AAGN_5_CTT), presumably as a homodimer. TRD1 alone (allele variant 3) did not modify a palindromic version of its half-site motif (CTTTAN_7_TAAAG) as potentially expected, perhaps because the helical connector could not form a homodimer (Figure 4C). According to the current nomenclature, JHP1423 was named M.Hpy99XV.

#### JHP1272

**Figure 5.**
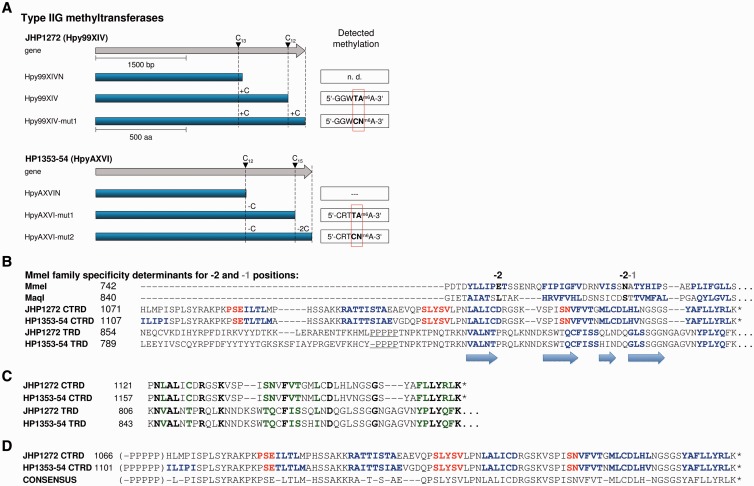
Graphical representation of the specificity switching phenomenon of the two homologous Type IIG MTases JHP1272 and HP1353-HP1354. (**A**) The gene sequences (gray arrows) contain two putative or authentic frameshifts that would prevent translation of full-length proteins (blue bars). Each frameshift was repaired through site-directed mutagenesis [addition or deletion of C nucleotide(s) is indicated] and correction was verified by targeted Sanger sequencing. Plasmid DNA (Hpy99XIV) or gDNA (remainder) of the *E. coli* host strains ER2796 was subjected to SMRT sequencing and analyzed for methylation. ‘mut’ marks alleles with frameshift corrections, ‘n.d.’ points out that no SMRT sequencing was performed and dashed lines indicate that no methylation could be detected. Filled inverted triangles highlight the position of the frameshift mutations. JHP—ORF of J99, HP—ORF of 26695. (**B–D**) Portions of the amino-acid multiple sequence alignments including secondary structure predictions (raw output of PROMALS) of the MTases JHP1272 and HP1353-HP1354. The numbers preceding each sequence display the position within the amino acid sequence. An asterisk at the end of a sequence indicates a stop codon. CTRD—C-terminal additional recognition domain, TRD—target recognition domain. (B) Secondary structure-guided alignment of JHP1272 and HP1353 CTRD and TRD with two MmeI family enzymes. The alignment shows the similarity of both MTases to the secondary structure of MmeI and MaqI in the MmeI region known to specify recognition of the −2 and −1 bases (relative to the methylated base within a recognition site). For these positions a changing specificity in the two active variants of JHP1272 and HP1353-HP1354 was observed. The red color text indicates predicted alpha helices and blue indicates predicted beta strands. The arrows indicate the four beta strands forming the structural motif in MmeI family enzymes that present −2 and −1 contact amino acids. The underlined PPPP region represents the first homopolymeric repeat of C nucleotides. (C) Putative recognition region for −1 and −2 base positions within the regular TRD and CTRD of the two MTases. Amino acid identities are depicted in bold black, similarities in bold green. (D) Alignment of HP1353-HP1354 and JHP1272 CTRD. Both regions show 87% amino acid identity (82 of 94), which most likely accounts for the identical specificity switch for −1 and −2 base positions observed for both protein variants containing the CTRD.

The gene of the Type IIG system JHP1272 of strain J99 was reported to contain two homopolymeric nucleotide repeats, which result in two frameshifts (21) (Figure 5A), suggesting that JHP1272 is inactive in strain J99. In strain J99-R3, the first repeat is one base pair longer and in frame, so that JHP1272 encodes a 1071 amino acid protein. Expression of this variant in *E. coli* and SMRT sequencing of the plasmid DNA assigned GGWTAA methylation to JHP1272 (Figure 5A and Table 2). In fact, we observed methylation of the more degenerate sequence GGWTRA (W—A or T, R—A or G) in the *E. coli* background, but this is most likely due to overexpression of the MTase on a high-copy plasmid (∼200 copies/cell). This hypothesis is supported by the methylome data of J99-R3, for which occasional methylation of GGWTGA could be detected by manual data analysis (12% of GGWTGA sites detected as methylated), while the methylation rate for the GGWTAA sites was 99.3%. This system encoded by JHP1272, with the first frameshift repaired, was designated Hpy99XIV.

#### JHP1409

The Type IIG MTase gene JHP1409 was overexpressed in *E. coli* and the resulting plasmid was subjected to SMRT sequencing. This identified the JHP1409 recognition site as GCCT^m6^A, with methylation occurring only on the top strand (Table 2). It was referred to as Hpy99XIII and is an ortholog of HP1517.

**Table 3.** Comparison of methylation patterns between *H. pylori* 26695 and J99-R3

Specificity^a^	R-M characteristics			*H. pylori* 26695		*H. pylori* J99-R3		Nomenclature	
	Modification	Type	Sub type	Locus M gene^b^	Locus S gene^b^	Locus M gene^b^	Locus S gene^b^	26695^b^	J99-R3^b^
Specificities found in 26695 and J99-R3								
C**A**TG	^m6^A	IIP	Alpha	hp1208	–	jhp1131	–	M.HpyAI	M.Hpy99X
G**A**TC	^m6^A	IIP	Beta	hp0092	–	jhp0085	–	M.HpyAIII	M.Hpy99VI
TCG**A**	^m6^A	IIP	Beta	hp0260	–	jhp0244	–	M.HpyAX	M.Hpy99VII
G**A**NTC	^m6^A	IIP	Beta	hp1352	–	jhp1271	–	M.HpyAIV	M.Hpy99IX
ATTA**A**T	^m6^A	IIP	Gamma	hp0478	–	jhp0430	–	M.HpyAVII	M.Hpy99XIX
G**C**GC	^m5^C	IIP	5mC	hp1121	–	jhp1050	–	M.HpyAVIII	M.Hpy99III
G**A**GG	^m6^A	IIS	Beta	hp0050	–	jhp0043	–	M1.HpyAVI	M1.Hpy99V
Unique specificities of 26695								
GAAG**A**	^m6^A	IIS	Beta	hp1367	–	No homologue	–	M1.HpyAII	–
T**C**TTC	^m4^C	IIS	Beta	hp1368	–	No homologue	–	M2.HpyAII	–
**C**CTC	^m5^C	IIS	5mC	hp0051	–	jhp0044	–	M2.HpyAVI	M2.Hpy99V, inactive
GCGT**A**	^m6^A	IIG	Gamma	hp1517	–	(jhp1409)	–	HpyAXIV	(Hpy99XIII)
GA**A**GG/C**C**TTC	^m6^A, ^m5^C	IIS	Gamma,5mC	hp0054	–	No homologue	–	M.HpyAV	–
GC**A**G	^m6^A	III	Beta	hp0593	–	No homologue	–	M.HpyAXI	–
AC**A**N_8_TAG	^m6^A	I	Gamma	hp0850	hp0790	(jhp0786)	–	M.HpyAXIII	(M.Hpy99XVII)
CGR**A**T	^m6^A	?	?	?	–	–	–	–	–
Unique specificities of J99-R3								
GT**A**C	^m6^A	IIP	Gamma	(hp0503P)	–	jhp0454	–	M.HpyAXII, inactive	M.Hpy99XII
**C**CGG	^m4^C	IIP	Beta	(hp0263P)	–	jhp0248	–	Inactive	M.Hpy99VIII
TCNNG**A**	^m6^A	IIP	Gamma	(hp0369P)	–	jhp1012	–	Inactive	M.Hpy99XVIII
**C**CNNGG	^m4^C	IIP	Beta	No homologue	–	jhp0629	–	–	M.Hpy99IV
GTS**A**C	^m6^A	IIP	Beta	No homologue	–	jhp0045	–	–	M.Hpy99II
CGW**C**G	^m4^C	IIP	Beta	No homologue	–	jhp0756	–	–	M.Hpy99I
A**C**GT	^m5^C	IIP	5mC	(hp0483 fs)	–	jhp0435	–	Inactive	M.Hpy99XI
GCCT**A**	^m6^A	IIG	Gamma	(hp1517)	–	jhp1409	–	(HpyAXIV)	Hpy99XIII
GGWTA**A**	^m6^A	IIG	Gamma	(hp1353-mut1)	–	jhp1272	–	(HpyAXVI-mut1)	Hpy99XIV
RT**A**YN_5_RTAY	^m6^A	I	Gamma	(hp0850)	–	jhp0786	jhp0785	(M.HpyAXIII)	M.Hpy99XVI
A**A**GN_6_CTC	^m6^A	I	Gamma	(hp0850)	–	jhp0786	jhp0726	(M.HpyAXIII)	M.Hpy99XVII
A**A**GN_5_CTT	^m6^A	I	Gamma	No homologue	–	jhp1423	jhp1422	–	M.Hpy99XV
A**A**GN_6_TAAAG	^m6^A	I	Gamma	No homologue	–	jhp1423	jhp1422	–	M.Hpy99XV
Specificities found in 26695 following frameshift repair of the corresponding genes								
GGANN**A**G	^m6^A	ISP	Gamma	hp0669 (hp0668)	–	(jhp0612P)	–	M.HpyAXVIII	inactive
CRTTA**A**	^m6^A	IIG	Gamma	hp1353-mut1	–	(jhp1272)	–	HpyAXVI-mut1	(Hpy99XIV)
CRTCN**A**	^m6^A	IIG	Gamma	hp1353-mut2	–	(jhp1272-mut1)	–	HpyAXVI-mut2	(Hpy99XIV-mut1)
TC**A**G	^m6^A	III	Beta	hp1370	–	No homologue	–	M.HpyAXVII	–
Specificities found in J99-R3 following frameshift repair of the corresponding genes								
TC**A**N_6_TRG	^m6^A	IIB	Gamma	(hp1472)	(hp1471)	jhp1365	jhp1364	Inactive	Hpy99XXII
GWC**A**Y	^m6^A	III	Beta	(hp1522)	–	jhp1411	–	Inactive	M.Hpy99XXI
GGWCN**A**	^m6^A	IIG	Gamma	(hp1353-mut2)	–	jhp1272-mut1	–	(HpyAXVI-mut2)	Hpy99XIV-mut1
Silenced specificity found in J99-R3								
AA**A**N_6_TGG	^m6^A	I	Gamma	(hp0463P)	–	jhp0415	jhp0414	Inactive	M.Hpy99XX

^a^The methylated position within the motif is highlighted in bold. If present, underlining indicates the modified base in the complementary strand.

^b^Loci and systems displayed in brackets are homologous but have different specificities. The addition of a ‘P’ indicates that a gene is inactive. The locus hp0483 is inactivated by an authentic frameshift (fs).

### Analysis of putative phase variable genes

The genomes of *H. pylori* contain a number of genes with frameshift mutations often induced by homopolymeric nucleotide repeats. These repeats are prone to length changes due to slipped strand mispairing and can enable rapid phase variation. Among these putative or confirmed phase variable genes are several MTase genes, which are predicted to be inactive in the wild-type strains. We sought to correct the frameshifts within multiple MTase genes to analyze the activity of the resulting enzymes (see ‘Materials and Methods’ section). Using this approach, we could show activity for four of seven so far uncharacterized phase variable MTases that are inactive in the wild-type genome, and, in this process, identified a further novel feature of MTases.

#### Activity and specificity switching in Type IIG R-M systems

The two homologous Type IIG systems JHP1272 (J99-R3) and HP1353-HP1354 (26695) each contain two homopolymeric repeats that can lead to premature stop codons. In J99-R3, the first homopolymeric repeat in JHP1272 was naturally in frame, and the enzyme was consequently active (GGWTAA). Unexpectedly, repairing the second frameshift altered the recognition site of the enzyme to GGWCNA (Figure 5A). JHP1272 wt (Hpy99XIV) catalyzed the complete modification of GGWTAA in the J99-R3 genome and partial modification of GGWTGA. Both sequences were completely modified when JHP1272 wt was overexpressed in *E. coli*, while the full-length protein JHP1272-mut1 modified GGWCNA (92.3% detected) and GGWTAA (72.8% detected) in the *E. coli* genome. A similar frameshift-mediated switching of recognition specificity was demonstrated for HP1353-HP1354 where correction of the first frameshift (mut1 allele) activated the gene and resulted in methylation of the site CRTTRA (R—A or G) when overexpressed in *E. coli* (96.8% CRTTAA detected, 39.9% CRTTGA detected), which further changed to CRTCNA when the second frameshift was also repaired (mut2 allele: 98.6% CRTCNA detected; CRTTRA not detected).

For both Type IIG systems, repair of the second frameshift appeared to alter specificity at the −1 and −2 position relative to the modified A nucleotide (Figure 5A). These remarkable enzymes are the first example of Type IIG restriction genes that can change specificity simply by adding a small additional domain. The additional domain has significant sequence similarity to a portion of the TRD within the shorter, normal length variant (Figure 5C). This similar region has predicted secondary structure similar to that observed in the TRD region of the MmeI family that has been shown to recognize the −2 and −1 base pairs (Figure 5B), i.e. four short beta strands with recognition amino acids positioned at the turn between strands 1 and 2, and between strands 3 and 4 (40). It thus appears that this additional domain displaces or substitutes for the portion of the regular TRD, specifying recognition of the −2 and −1 base pair positions, thereby changing recognition at these two positions. In the case of HP1353-HP1354, recognition appears to cleanly change to the new CRTCNA specificity, while for JHP1272, at least in the context of overexpression in *E. coli*, the new specificity is preferred but not exclusive (92.3% GGWCNA detected and 72.8% GGWTAA detected). These genes thus have two switches: the first homopolymeric switch turns the gene on and off, while the second changes the recognition specificity (Figure 5D).

The enzymatically active form of JHP1272 with the first repeat naturally in frame (as detected in J99-R3) was named Hpy99XIV, while the allele variant with two repaired frameshifts was named Hpy99XIV-mut1. For 26695, HP1353-HP1354-mut1 was designated HpyAXVI-mut1 and the mut2 allele variant HpyAXVI-mut2 (see also Figure 5A).

Purified full-length JHP1272 was active as an endonuclease. The limited quantity of enzyme purified produced a restriction digestion pattern consistent with GGWCNA recognition; however, the pattern was not stable with increasing amounts of enzyme, indicating further cutting at the GGWTAA recognition motif of the wt enzyme, which is consistent with the observed methylation of both GGWCNA and GGWTAA. HP1353-HP1354 enzyme has not been isolated and tested for endonuclease activity.

#### Phase variable Type III MTases

We next assessed the activity and specificity of three putative phase variable Type III MTases (26695: HP1369-HP1370, HP1522; J99-R3: JHP1411) and could reactivate HP1369-HP1370 and JHP1411 but not HP1522 through frameshift correction (Figure 2B). HP1369-HP1370 catalyzes methylation of the novel recognition site 5′-TC^m6^AG-3′. In 26695, a frameshift near the 3′-end of HP1369 causes a premature stop codon and splits the gene into two ORFs. Consistently, methylation of TCAG was not detected in 26695 (Figure 1A), and functional inactivation of the individual ORFs had no effects on the methylome (Supplementary Table S5). Correction of the JHP1411 frameshift resulted in an active MTase, which methylates the novel sequence 5′-GWC^m6^AY-3′ (W—A or T, Y—C or T) (Figure 2B), whereas enzyme activity could not be restored for the homologous system HP1522. Like all known Type III MTases, JHP1411 and HP1369-HP1370 only modify one DNA strand. According to the current nomenclature we refer to the active MTases HP1369-HP1370 as M.HpyAXVII and JHP1411 as M.Hpy99XXI.

#### BcgI-like systems

The BcgI-like systems HP1471/HP1472 and JHP1364/JHP1365 belong to the Type IIB family of MTases. The heterooligomeric enzymes consist of an S subunit (HP1471, JHP1364) and a gene fusion of an MTase and a REase (RM gene; HP1472, JHP1365). Both systems were reported to be inactive (22,23). This is most likely due a stop codon downstream of the RM gene without a potential start codon to reinitiate transcription of the S subunit. Furthermore, the two S subunit genes are prone to phase variation due to the presence of a homopolymeric G repeat (Figure 2C). In the published genome sequences of 26695 (19) and J99 (21), repeat lengths of 14 nucleotides have been reported, not resulting in a frameshift. The respective strains in our laboratory, however, harbor repeats of 13 G nucleotides, which prevent full translation of HP1471 and JHP1364. To characterize the activity of these systems, we altered the sequence between the RM gene and the S subunit gene to provide an optimal ribosomal binding site in *E. coli* and a start codon, and corrected the frameshift within the S subunit gene. Expression of the modified JHP1364/JHP1365 system produced adenine modification at the novel recognition site TC^m6^AN_6_TRG (R—A or G) (Figure 2C). This system was designated Hpy99XXII. We could not detect base modification for the corrected HP1471/HP1472 system (Figure 2C).

### Type ISP systems

Type ISP systems are characterized by the joint expression of sequence specificity, methylation, translocation and restriction units in a single polypeptide (SP) (44). The 26695 genome contains the gene cluster HP0667-HP0668-HP0669, which together could encode a Type ISP system. Two frameshifts (not due to sequence repeats) prevent expression of a full-length protein (Figure 2D). Correction of the first or both frameshifts resulted in expression of an active ^m6^A MTase that catalyzed methylation of the novel recognition site 5′-GGANN^m6^AG-3′. In addition, overexpression of HP0669, which includes the MTase and specificity domains, revealed that HP0669 alone is an active MTase in *E. coli* that recognizes the same GGANNAG motif (Figure 2D). Thus we named the product of HP0669 as M.HpyAXVIII.

JHP0612-JHP0613 is the homologous Type ISP system of strain J99-R3, which shows 86% amino acid identity to HpyAXVIII. It contains one authentic frameshift, but correction of the mutation did not result in an active MTase (Figure 2D).

## DISCUSSION

This project was initiated because we were intrigued by the recent demonstration that SMRT sequencing permits the genome-wide identification of methylated bases in bacteria. Since *H. pylori* displays extensive diversity of its content of R-M systems between different strains, which may have important functional consequences for DNA uptake, genome diversification and gene regulation, SMRT sequencing offered the potential to study DNA methylation in this pathogen at a genome-wide scale.

### *Helicobacter pylori* genomes display dense and strain-specific methylation

We determined the methylomes of the well-characterized *H. pylori* strains 26695 and J99-R3 by SMRT sequencing, identifying 17 and 22 methylated sequence patterns, respectively. Only seven of these were detected in both strains, while the remaining methylation patterns resulted from strain-specific MTase activities.

Our study is the first to analyze all three types of methylation using SMRT sequencing. The detection of ^m4^C and ^m6^A modifications can be achieved by computational analysis of SMRT sequence data from untreated DNA samples because these modifications affect base pairing due to their position and therefore have a strong effect on polymerase kinetics. In contrast, ^m5^C methylation has a smaller impact on the polymerase because it is orientated toward the major groove (26). Recently, Tet1-mediated conversion of ^m5^C to the larger modification ^5ca^C (34,45) has been used to enhance the magnitude of the kinetic signal, thus increasing the interpulse duration ratio for ^m5^C methylation (46). Using this approach, we could confirm the previously described ^m5^C MTase activities of both *H. pylori* strains (22,24).

Both analyzed *H. pylori* strains showed dense methylation throughout most of the genomes. In both strains, we noted a comparatively lower methylation density in a region previously identified as a plasticity zone (PZ) (21), which was most likely acquired from other bacterial species via horizontal gene transfer. The mean density of methylated sites in the PZs of 26695 and J99-R3 is 24.1 and 17 motifs per kb, respectively, compared with 34.6 and 34.8 methylated motifs per kb for the remainder of the genomes. In contrast, the *cag* pathogenicity island (*cag*PAI) of *H. pylori*, which codes for a major virulence module (47), showed a slightly higher density of methylated sites compared with the remainder of the genome (26695: 36.1 versus 34.1 motifs per kb; J99-R3: 35.7 versus 34.3). The difference in methylation density between PZ and *cag*PAI was not correlated with the estimated time of acquisition by horizontal gene transfer because many of the genes within the PZ are likely to have been acquired before the *cag*PAI (48).

### From novel methylated recognition sites to novel *H. pylori* MTases

Methylome profiling through SMRT sequencing permitted the identification of numerous novel methylated sequence patterns in both *H. pylori* strains. The combined strategy of allelic disruption of candidate genes and additional functional tests led to the identification of new MTase activities in *H. pylori* responsible for methylation of eight of the nine novel recognition sites and, furthermore, to the description of unexpected new features of R-M systems.

### Type I R-M systems with multiple specificities

In this study, we characterized several Type I MTases with multiple specificities (JHP1422/JHP1423; JHP0785/JHP0786 and JHP0726). Type I systems are typically a challenge for characterization due to their complex composition. Their restriction and modification subunits are both dependent on an S subunit, which mediates DNA recognition (49). It was commonly accepted that the S subunit is composed of two TRDs, which are either homologous (both TRDs are identical) or nonhomologous, resulting in the recognition of either palindromic or nonpalindromic recognition sequences, respectively. Our study adds an additional element to this paradigm. The functional characterization of JHP1422, which encodes an S subunit with three TRDs, demonstrates the ability of a single S subunit to recognize both one palindromic and one nonpalindromic recognition site. Sequence comparisons to other R-M genes in the REBASE database revealed no strong homolog of JHP1422 that also contains three TRDs although there are several S subunits that are predicted to contain three TRDs. Homologs coding for S subunits with two TRDs similar to TRD1 and TRD2/3 of JHP1422 (ORF703 of *H. pylori* FD703) can be found by similarity searching. The tripartite JHP1422 could have arisen through an intrachromosomal duplication, as proposed by Kobayashi and coworkers (50), resulting in an unusually long S subunit protein that evolved to recognize different target sequences. This hypothesis is supported by the fact that we observed allelic variation of JHP1422 even within the J99-R3 gDNA used for SMRT sequencing.

The MTase JHP0786 can achieve specificity from either its adjacent S subunit JHP0785 or the orphan S subunit JHP0726. To our knowledge, this is the first report where an MTase can assume a new specificity through interaction with an alternative S subunit located distantly on the chromosome. JHP0785 encodes only one TRD and we propose that a homodimer of JHP0785 could form an active S subunit. This assumption would be in agreement with earlier studies on HsdS proteins, which showed that after truncation, TRD1 or TRD2 (plus adjacent conserved regions) are each sufficient for recognition of a palindromic site (51,52). Further investigation is needed to disclose how the two S subunits compete for association with JHP0786, although it seems likely that the interaction is enabled by sequence homologies in certain regions of the two HsdS subunits.

The Type I MTase HP0850 of strain 26695 is also able to associate with the orphan S subunit HP0790. Its adjacent S subunit HP0848-HP0849 is most likely inactivated by a confirmed frameshift. So far, it is not known whether correction of the mutation would result in an active S protein, which, in association with HP0850, could add another Type I recognition site to the methylome of 26695.

### Frameshift-mediated switching of MTase sequence specificity

As a further intriguing novel feature, we identified a mechanism of specificity switching that depends on the presence or absence of an extra domain on the C-terminus of the Type IIG MTases JHP1272 and HP1353-HP1354. This has a major impact on the bioinformatic assignment of specificities to predicted MTase genes. The predictions of MTase specificities are based on sequence homologies with characterized enzymes but it has been shown that the exchange of two amino acids, and in some cases of even a single amino acid in critical regions, e.g. in the DNA sequence recognition domain, can change the recognition sequences of the mutated enzyme (40). The exact molecular mechanism of the specificity switching remains to be identified. JHP1272 and HP1353-HP1354 show significant similarity to other Type IIG R-M systems, such as TaqII or MmeI. Type IIG systems combine methylation and restriction activity in a single protein and catalyze hemi-methylation of the DNA. To accomplish protection against endonuclease cleavage, some Type IIG families must partner with a second MTase to achieve methylation of both DNA strands (53). In contrast to that, hemi-methylation of the DNA by members of the MmeI family and many other Type IIG enzymes is sufficient for host protection; these systems are termed Type IIL enzymes (i.e. L for Lone-strand modification) (54). An assignment of both *H. pylori* MTases to the Type IIL family is suggestive because their recognition motif is modified on only one DNA strand in *H. pylori* gDNA and we could express JHP1272 (which also encodes an active endonuclease domain) without a partner Type IIG system. In this context it is noteworthy that switching of the JHP1272 MTase specificity from GGWTAA to GGWCNA could be deleterious for cells that express an active JHP1272 REase unless the specificities of both enzyme activities switch simultaneously. Since the DNA recognition, which targets both methylation and restriction activities within Type IIL systems, is mediated by a shared C-terminal TRD, alteration in the MTase specificity also results in an altered recognition site for the restriction domain. This was demonstrated *in vitro* for the MmeI family by Morgan and Luyten who showed that amino acid substitution of critical positions within the TRD result in a new recognition site targeted by the MTase and REase domain (40).

### A new family of Type IIG MTases

The homologous MTases JHP1409 (Hpy99XIII) and HP1517 (HpyAXIV) are both active and catalyze methylation of the similar recognition sites GCCTA and GCGTA, respectively. A third closely related homolog, CjeFV of *Campylobacter jejuni* strain 81-176, methylates the sequence GGRC^m6^A (28). All three proteins represent a potentially new family of Type IIG R-M systems, the CjeFV family. The three MTases bear some sequence similarity to the CjeFIII and Eco57I Type IIG families. However, important differences suggest the CfeFV family is distinct from both of those: CjeFIII family members have a 6-bp recognition site with methylation on an internal A residue, whereas CjeFV has a 5-bp recognition site with methylation on the terminal A. Eco57I members have an accompanying MTase gene, whereas CjeFV members do not. Homologs of JHP1409 and HP1517 are surprisingly well conserved within Epsilonproteobacteria, with apparent orthologs in most *H. pylori* strains as well as *Helicobacter acinonychis* and *Helicobacter mustelae*, and a highly related set of orthologs in most *Campylobacter jejuni* strains as well as *Campylobacter lari*.

### Results of methylome analysis in the context of previous reports about *H. pylori* R-M systems

The majority of Type II R-M systems of *H. pylori* strains 26695 and J99 had been functionally characterized previously (22,23). Our methylome analysis was in agreement with most of these earlier findings, but three conflicting results were obtained. In contrast to the earlier publications, methylation of their putative recognition sites suggested activity of JHP0430 and JHP1012 (22), while HP0910 (23) appeared to be inactive. Subcloning of the MTases confirmed the data obtained by SMRT sequencing. There are different possible reasons for the observed conflicts. Strain J99 used by Kong and colleagues (22) may have acquired loss-of-function mutations in genes JHP0430 and JHP1012. Furthermore, the dot blot assay used for testing MTase activity with ^m6^A specific antibodies might have yielded false-negative results or the expression plasmid acquired mutations impeding expression of the MTases. For 26695, we could not detect methylation of GTNNAC by HP0910 through SMRT sequencing. Restriction experiments with 26695 gDNA and overexpression of HP0910 confirmed that the putative MTase does not methylate GTNNAC in our hands. This result is in agreement with the earlier observation that 26695 gDNA was sensitive to restriction with Hpy8I, which cleaves GTNNAC (24).

### Phase variation of MTases in *H. pylori*

The *H. pylori* genomes are rich in homopolymeric tracts or dinucleotide repeats (19,55), which can mediate phase variation through slipped-strand mispairing, allowing rapid adaptation to environmental changes via reversible ON/OFF switching of gene expression (3). Gene regulation via phase variation has been described for many *H. pylori* genes, including the outer membrane protein genes *hopZ* (56), *babA* (57,58) and *sabA* (59,60), the *fliP* gene encoding a flagellar basal body protein (61), and also for Type III MTases (62,63). *Helicobacter pylori* 26695 and J99-R3 contain several putative phase variable MTase genes. The homologs JHP1272 and HP1353-HP1354 are switched on and off through phase variation at their first homopolymeric tract in addition to their novel specificity switching through the second homopolymeric tract. In total, we could restore the activity of four so far uncharacterized *H. pylori* enzymes in an *E. coli* host, while two systems could not be reactivated through frameshift repair. Whether similar reactivation of some of these enzymes also occurs in *H. pylori* remains to be investigated, although this seems likely, at least in the case of long (>8 bp) homopolymeric repeats (55).

Our observation that GGWTAA methylation, catalyzed by JHP1272 with the first frameshift repaired, was found for J99-R3 and 9 of 10 isogenic mutant strains, is a strong indication that phase variation of MTases also occurs in *H. pylori*. The remaining mutant strain showed methylation of the motif GGWCNA, the specificity of full-length JHP1272, but not GGWTAA. Sequence analysis of this mutant strain revealed the presence of the JHP1272-mut1 variant in which the length of both homopolymeric tracts was naturally corrected. The strong selection of the two active JHP1272 variants is of biological relevance for J99-R3, since the endonuclease domain of both variants, encoded in the N-terminal region of the gene, was found to be active in *E. coli*.

What is the biological role of these phase variable MTases? Phase variation of surface-associated proteins leads to generation of a heterogeneous population that expresses a different set of genes allowing rapid adaptation to different environmental conditions (3,64). The reversible ON/OFF switching of MTase genes may result in analogous heterogeneity in the methylation patterns. Thus, phase variation might provide subpopulations with a barrier to phage infection or represent a mechanism to regulate genetic exchange between unrelated strains.

An impact of phase variable MTases on the expression of multiple genes was described for pathogenic bacteria such as *Haemophilus influenzae* (65), *Neisseria spp.* (66) and also for *H. pylori*. The term ‘phasevarion’ has been coined to describe this phenomenon (65,67). A recent study showed that a phase variable Type III MTase of *H. pylori* P12 (HPP12_1497) affected the expression of six genes (63). However, the activity of HPP12_1497 was only indirectly shown and its target site remains to be identified (63). Homologs of this enzyme are also found in 26695 (HP1522) and J99 (JHP1411), and in our hands, JHP1411 but not HP1522 was active after adjusting the repeat length. The frequency and potential role of changes of R-M system activity during infection remain poorly understood and need further investigation.

## CONCLUSION

In summary, we found that the *H. pylori* genome is highly methylated, and methylation patterns differ widely between strains. We further showed that this pathogen is not only remarkable due to its abundance of active MTases but also harbors R-M systems with exceptional versatility. We report methylation of two distinct recognition sites by one Type I MTase, either by association with a tripartite S subunit or by interaction with two alternative S subunits, one of which is encoded remotely. As a further intriguing novel feature, we identified homologous Type IIG systems with two phase variable sites conferring an ON/OFF switch and a specificity switch that depends on the presence or absence of an extra domain at the C-terminus. The data show that R-M systems are more complex than previously thought and with the further use of SMRT® sequencing for the discovery and functional characterization of R-M systems, our knowledge is likely to rapidly increase. The methylomes of the well-characterized strains 26695 and J99-R3 will provide a valuable resource for future studies investigating the role of R-M systems in genetic diversification of *H. pylori* as well as the impact of DNA methylation on gene expression and host interaction.

## SUPPLEMENTARY DATA

Supplementary Data are available at NAR Online, including [68–74].

## FUNDING

German Research Foundation (DFG) [DFG SFB 900/A1 and DFG SFB 900/Z1 to S.S.]; DFG [DFG SFB 900/B6] and BMBF (HELDIVPAT) in the framework of the ERA-NET PathoGenoMics (to C.J.); German Center of Infection Research (DZIF) [8000-105-3 to J.O.]; NIH [1R44GM105125 and 1R44GM100560 to R.J.R.]; PhD stipend from the DFG (to J.Kr.) in the framework of the International Research Training Group IRTG 1273. Funding for open access charge: German Research Foundation.

*Conflict of interest statement*. K.L., C.K. and J.Ko. are full-time employees at Pacific Biosciences, a company commercializing SMRT sequencing technologies. R.D.M., B.P.A. and R.J.R. are full-time employees of New England Biolabs, a company that sells research reagents such as DNA MTases.

## Supplementary Material

Supplementary Data
