# Supplementary material for: The complex methylome of the human gastric pathogen Helicobacter pylori
Source: Nucleic Acids Res. 2013 Dec 2;42(4):2415–32. doi: 10.1093/nar/gkt1201 (PMC3936762; doi:10.1093/nar/gkt1201)
Supplement: Supplementary Data [file supp_42_4_2415__index.html]

The complex methylome of the human gastric pathogen Helicobacter pylori — The complex methylome of the human gastric pathogen Helicobacter pylori — Supplementary Data 

# The complex methylome of the human gastric pathogen *Helicobacter pylori*

## Supplementary Data

files

**Files in this Data Supplement:**

- Supplementary Data - pdf file
