# Supplementary material for: The complex methylome of the human gastric pathogen Helicobacter pylori
Source: Nucleic Acids Res. 2013 Dec 2;42(4):2415–32. doi: 10.1093/nar/gkt1201 (PMC3936762; doi:10.1093/nar/gkt1201)
Supplement: Supplementary Data [file supp_gkt1201_nar-02606-h-2013-File007.pdf]

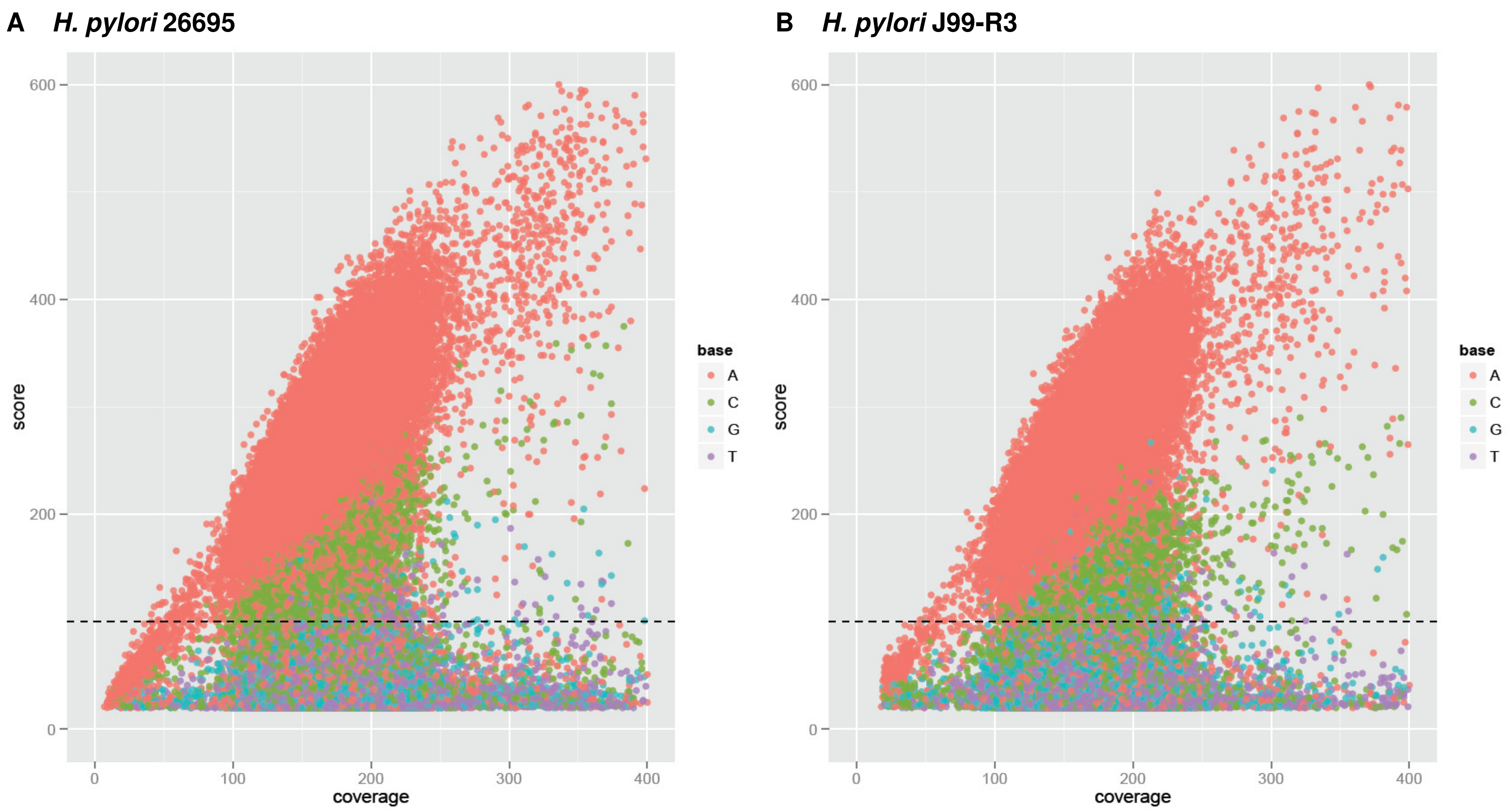

Krebes et al., Figure S1

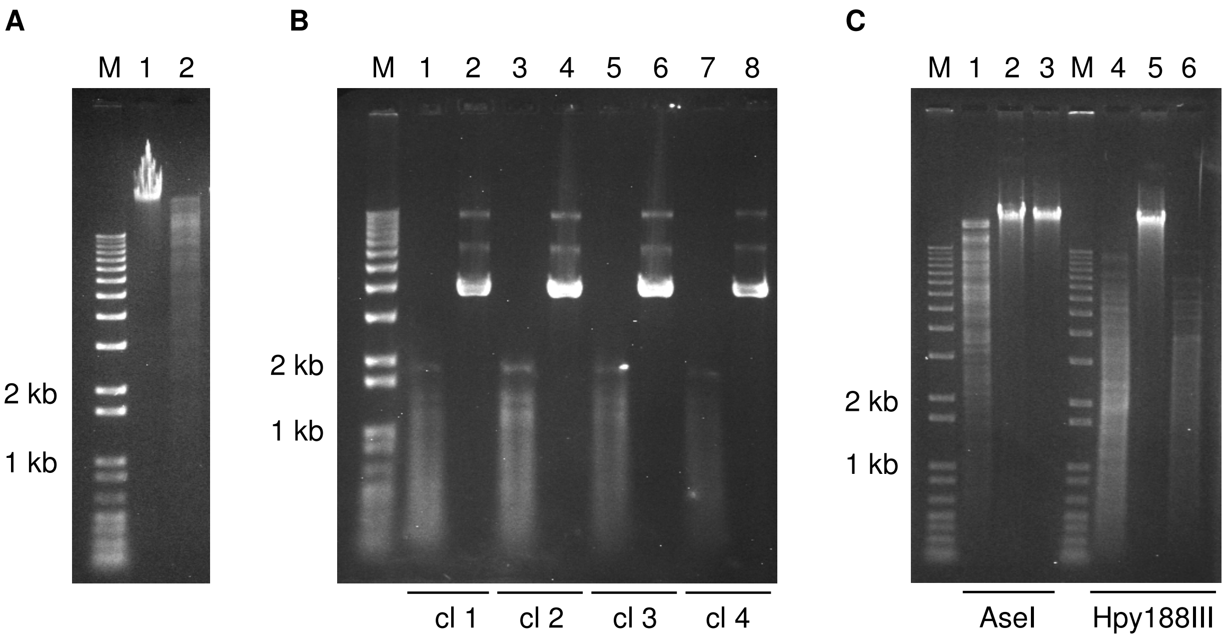

Krebes et al., Figure S2

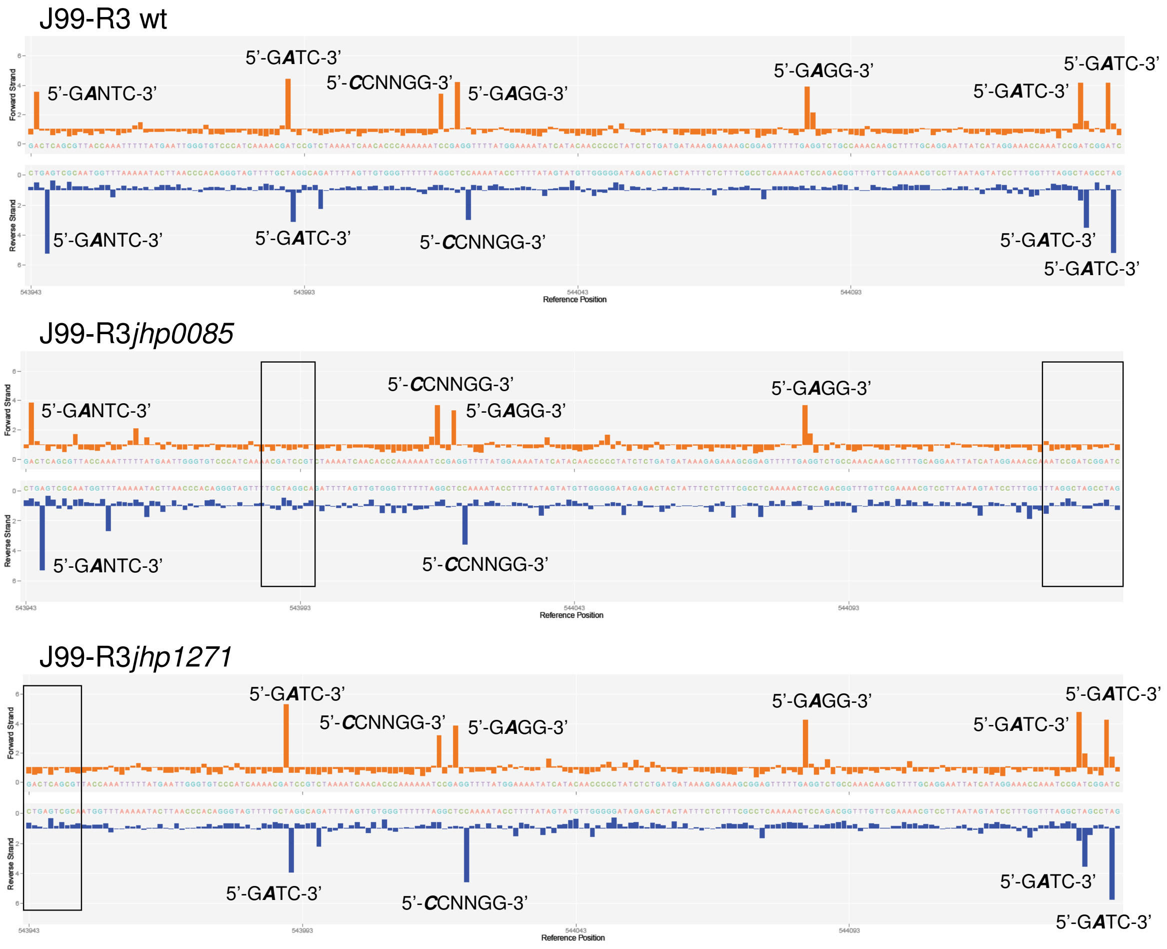

Krebes et al., Figure S3

Legends for Supplementary Figures

Figure S1: Scatter plots of sequencing coverage (x-axis) and kinetic score (y-axis) for all genomic positions of *H. pylori* strains 26695 (**A**) and J99-R3 (**B**). The bases can be differentiated via the colour-coding depicted on the upper left part of each plot. The dashed line represents the cutoff for detection of methylated genomic positions.

Figure S2: Analysis of MTase activity by DNA restriction experiments. **A.** 300 ng of 26695 gDNA was either incubated w/o restriction enzymes (lane 1) or with Hpy166II (lane 2), an isoschizomer of HP0909, confirming that HP0910 is inactive in the genome. **B**. The sensitivity of the plasmid DNA (pSUS3071) containing a copy of HP0910 to cleavage by Hpy166II was tested. The HP0910 gene was expressed via the strong *ureA* promoter. MTase activity of four independent clones (cl 1 - 4) was assayed by incubation of 200 ng plasmid DNA with Hpy166II (lanes 1, 3, 5, and 7) and w/o enzyme (lanes 2, 4, 6, and 8); expression of HP0910 did not prevent cleavage. **C.** Restriction digestion of mutant and wild type gDNA confirms lack of gene activity of the JHP0430 and JHP1012 mutant strains. 300 ng of gDNA were incubated with either AseI (isoschizomer to JHP0431) or with Hpy188III (isoschizomer of JHP1013). Lane 1: J99-R3*jhp0430*, lane 2 and 5: J99-R3, lane 3 and 6: 26695, lane 4: J99-R3*jhp1012*. Lane M, 1 kb Plus DNA Ladder, Invitrogen.

Figure S3: Kinetograms comparing the methylation pattern of a 200 bp region within the *cagA* gene in J99-R3 and its two isogenic mutant strains J99-R3*jhp0085* (lacks GATC methylation) and J99-R3*jhp1271* (lacks GANTC methylation). The genomic positions in bps are based on the J99 reference genome (21) and are displayed on the x-axis. The IPD ratios of each position in the forward (orange) and reverse strand (blue) are plotted on the y-axis. Motifs were assigned to each methylated position which was highlighted in bold. Black boxes indicate absent methylation patterns in the mutant strains.

Table S1: Bacterial strains.

Strain	Genotype	Source
Escherichia coli		
DH5α	F^-, φ80d*lacZ*ΔM15, Δ(*lacZYA-argF*)U169, *deoR*, *recA*1, *endA*1, *hsdR*17(rk^-, mk^+), *phoA*, *supE*44, λ^-, *thi*-1, *gyrA*96, *relA*1	(1)
ER2683	*fhuA2 glnV44 e14- rfbD1? relA1? endA1 spoT1? thi-1 Δ (mcrC-mrr) 114::IS10 Δ (lacI-lacA) 200/F'proAB* $lacI^q$ *ΔlacZM15 zzf::miniTn10 (KanR)*	(2)
ER2796 (=DB24)	*dam-16::Kan trp-31 his-1 fhuA2 rpsL104 D(lacZ)r1 glnV44 xyl-7 mtl-2 metR1 mcr-62 argG6 D(mcrB-hsd-mrr)114 dcm-6 zed-501::Tn10*	(3)
MC1061	*ara*D139, Δ(*ara*, *leu*)7697, Δ*lac*X74, $galU^-$, $galK^-$, hsr^-, hsm^-, *strA*	(4)
Helicobacter pylori		
26695	*H. pylori* wild type strain	(5)
J99-R3	Derivative of J99; A to T mutation at position 1618 in *rpoB*; Rif^r	(6)
26695*hp0462*	HP0462 (predicted type I R-M specificity protein) from wild type strain 26695 inactivated with *aphA-3*	This study
26695*hp00790*	HP0790 (predicted type I R-M specificity protein) from wild type strain 26695 inactivated with *aphA-3*	This study
26695*hp0848-49*	HP0848-49 (predicted type I R-M specificity protein) from wild type strain 26695 inactivated with *aphA-3*	This study
26695*hp0850*	HP0850 (predicted type I R-M modification protein) from wild type strain 26695 inactivated with *aphA-3*	This study
26695*hp1369*	HP1369 (predicted type III R-M modification protein) from wild type strain 26695 inactivated with *aphA-3*	This study
26695*hp1370*	HP1370 (predicted type III R-M modification protein) from wild type strain 26695 inactivated with *aphA-3*	This study
26695*hp1403*	HP1403 (predicted type I R-M system modification subunit) from wild type strain 26695 inactivated with *aphA-3*	This study
26695*hp1472*	HP1472 (predicted non-functional type IIS alpha subunit) from wild type strain 26695 inactivated with *aphA-3*	This study
26695*hp1517*	HP1517 (predicted type IIS restriction-modification protein) from wild type strain 26695 inactivated with *aphA-3*	This study
26695*hp1522*	HP1522 (predicted type III R-M modification protein) from wild type strain 26695 inactivated with *aphA-3*	This study
J99-R3*jhp0085*	JHP0085 (GATC site-specific type II adenine methyltransferase) from strain J99-R3 inactivated with *aphA-3*	This study
J99-R3*jhp0414*	JHP0414 (predicted type I R-M specificity protein) from strain J99-R3 inactivated with *aphA-3*	This study

J99-R3*jhp0430*	JHP0430 (probable type II adenine specific methyltransferase) from strain J99-R3 inactivated with *aphA-3*	This study
J99-R3*jhp0612*	JHP0612 (remnant of type I R-M polypeptide) from strain J99-R3 inactivated with *aphA-3*	This study
J99-R3*jhp0726*	JHP0726 (predicted type I R-M specificity protein) from strain J99-R3 inactivated with *aphA-3*	This study
J99-R3*jhp0785*	JHP0785 (predicted type I R-M specificity protein) from strain J99-R3 inactivated with *aphA-3*	This study
J99-R3*jhp0786*	JHP0786 (predicted type I R-M modification protein) from strain J99-R3 inactivated with *aphA-3*	This study
J99-R3*jhp1012*	JHP1012 (predicted non-functional adenine methyltransferase) from strain J99-R3 inactivated with *aphA-3*	This study
J99-R3*jhp1271*	JHP1271 (GANTC site-specific type II m6A methyltransferase) from strain J99-R3 inactivated with *aphA-3*	This study
J99-R3*jhp1284*	JHP1284 (predicted type III R-M modification protein) from strain J99-R3 inactivated with *aphA-3*	This study
J99-R3*jhp1296*	JHP1296 (predicted type III R-M modification protein) from strain J99-R3 inactivated with *aphA-3*	This study
J99-R3*jhp1365*	JHP1365 (predicted non-functional type IIS alpha-subunit) from strain J99-R3 inactivated with *aphA-3*	This study
J99-R3*jhp1423*	JHP1423 (predicted type I R-M modification subunit) from strain J99-R3 inactivated with *aphA-3*	This study

Table S2: Oligonucleotide primers used for *H. pylori* insertion mutagenesis.

Primer	Target gene	5' → 3' sequence	RS*	Application
hp0462_PstI_for	HP0462	atactgcagACGCGCTTAAGACCAAGCGA	PstI	Cloning
hp0462_BamHI_rev		ataggatccAGGAGATAACCTAACATGAGA	BamHI	
hp0462_XhoI_inv1		atactcgagACCTTGATTGGTGCATAGCCT	XhoI	Inverse PCR
hp0462_XhoI_inv2		atactcgagTCAACGAGCTTTTACACACGCT	XhoI	
hp0790_PstI_for	HP0790	atactgcagATGCATAAAATAGAGCGCTTAC	PstI	Cloning
hp0790_BamHI_rev		ataggatccTTACGCAAGCTCTTTGCTATTTAG	BamHI	
hp0790_BglII_inv1		ataagatctATCCACAGAAGCAAAACCTG	BglII	Inverse PCR
hp00790_BglII_inv2		ataagatctTGGAGCAAATGGAATTCAAG	BglII	
hp0848-49_PstI_for	HP0848-HP0849	atactgcagATAGAGCAACTGCTCCAAACTC	PstI	Cloning
hp0848-49_BamHI_rev		ataggatccTTATTCCTTGTTTTGGAGAGGT	BamHI	
hp0848-49_BglII_inv1		ataagatctCTATTCCTCCAAAACTTTTACT	BglII	Inverse PCR
hp0848-49_BglII_inv2		ataagatctGAATGTATCCAGTGATAAATTC	BglII	
hp0850_PstI_for	HP0850	atactgcagACCAAGCGTCATTAGAACGCA	PstI	Cloning
hp0850_BamHI_rev		ataggatccTGGATTCAAGACGGTTCCT	BamHI	
hp0850_XhoI_inv1		ataagatctTGAAGGAAAAACTCAAAGGC	XhoI	Inverse PCR
hp0850_XhoI_inv2		ataagatctATTTTAGATCCTAACACGCC	XhoI	
hp1369_PstI_for	HP1369	atactgcagATGAAAACGAACGAAGCGCA	PstI	Cloning
hp1369_BamHI_rev		ataggatccACACTTCGTCCATGAGCACT	BamHI	
hp1369_XhoI_inv1		atactcgagTGCTGCTGAGCGTAACGCTA	XhoI	Inverse PCR
hp1369_XhoI_inv2		atactcgagACACCATTAAGGAAGTGGCT	XhoI	
hp1370_PstI_for	HP1370	atactgcagAGTGCTCATGGACGAAGTGT	PstI	Cloning
hp1370_BamHI_rev		ataggatccAGCGTTGATCAAAGCTTGCT	BamHI	
hp1370_BglII_inv1		ataagatctAATGCGTTGGAGTAGGGCTT	BglII	Inverse PCR
hp1370_BglII_inv2		ataagatctAAGCGGTGCGGCTAAAGAA	BglII	
hp1403_PstI_for	HP1403	atactgcagATGGATGCGAGCGAGTATA	PstI	Cloning
hp1403_BamHI_rev		ataggatccAGAGCTGATACACGCCGTAT	BamHI	
hp1403_BglII_inv1		ataagatctTCACCGGCTAAACTAGAAGCT	BglII	Inverse PCR
hp1403_BglII_inv2		ataagatctTGCTCCACATCATCAAATCCT	BglII	
hp1472_PstI_for	HP1472	atactgcagAGTTCAATCTATTGATCCTTT	PstI	Cloning
hp1472_BamHI_rev		ataggatccATTGGATGATCACGCTCGCA	BamHI	
hp1472_BglII_inv1		ataagatctACATCTATCCCCATGCCTAGA	BglII	Inverse PCR
hp1472_BglII_inv2		ataagatctTCCAGCGATGAGGTCCATCA	BglII	
hp1517_PstI_for	HP1517	atactgcagAACAGCGAGCGCTTACCAGA	PstI	Cloning
hp1517_BamHI_rev		ataggatccAGATGTTAGGACATAGCGGT	BamHI	
hp1517_BglII_inv1		ataagatctTCCTCGCAACGCTTTTAGA	BglII	Inverse PCR
hp1517_BglII_inv2		ataagatctAGCTCAGGCTATGGATAGA	BglII	
hp1522_PstI_for	HP1522	atactgcagAAGCCCTTAAACGAATCCACTA	PstI	Cloning
hp1522_BamHI_rev		ataggatccCTACCCCCTAATCTTTAAATCG	BamHI	
hp1522_BglII_inv1		ataagatctTCAGATTTAACCCTAATACCT	BglII	Inverse PCR
hp1522_BglII_inv2		ataagatctAGGAAGACAAAAGCGCTTAT	BglII	

jhp0085_PstI_for	JHP0085	atactgcagTCGGTCTTTTTGTCCAAGCT	PstI	Cloning
jhp0085_BamHI_rev		ataggatccGTCAGCTTGGTATTGACTATC	BamHI	
jhp0085_BglII_inv1		ataagatctCGCTGATTAAAAGGCTTCCT	BglII	Inverse PCR
jhp0085_BglII_inv2		ataagatctACAAATGCGTGATGTGTGGA	BglII	
jhp0414_PstI_for	JHP0414	atactgcagTCACTATAGAAGACACGAGCGA	PstI	Cloning
jhp0414_BamHI_rev		atatctagaTGTCCGCAACGCCATTATACT	BamHI	
jhp0414_XhoI_inv1		atactcgagTCTTAACTTGGAATAGACCT	XhoI	Inverse PCR
jhp0414_XhoI_inv2		atactcgagTGGACAAATACGGAGAAGCT	XhoI	
jhp0430_PstI_for	JHP0430	atactgcagATGCCTTCAAACGCTCTTTCT	PstI	Cloning
jhp0430_BamHI_rev		ataggatccTTATCCCCTAAAGCCAAAAG	BamHI	
jhp0430_BglII_inv1		ataagatctAGGTTGAATTGCTGTTTGA	BglII	Inverse PCR
jhp0430_BglII_inv2		ataagatctTAGTGAGTTCAAACGCTCA	BglII	
jhp0612_PstI_for	JHP0612	atactgcagATGCAAGAAATCAGTGCCTA	PstI	Cloning
jhp0612_BamHI_rev		ataggatccTTAGTGCCTTTTGGCTTCAA	BamHI	
jhp0612_XhoI_inv1		atactcgagAAGGGGATTTCGTCTTCGGT	XhoI	Inverse PCR
jhp0612_XhoI_inv2		atactcgagTCTATCGTGTGCAGCGATGA	XhoI	
jhp0726_PstI_for	JHP0726	atactgcagAGAGTTATTACTCCACACTCT	PstI	Cloning
jhp0726_BamHI_rev		ataggatccTTAGGGGTTTGAAGGTCAGTA	BamHI	
jhp0726_XhoI_inv1		atactcgagACGATCTCTTGCTGGATCTCT	XhoI	Inverse PCR
jhp0726_XhoI_inv2		atactcgagAAGACAAGGGGAGACCTGTT	XhoI	
jhp0785_PstI_for	JHP0785	atactgcagATCTCGCCTTCACCATGCA	PstI	Cloning
jhp0785_BamHI_rev		ataggatccTGCTGTATTGGTGGATCACT	BamHI	
jhp0785_XhoI_inv1		atactcgagTTACTTCGCTCACTTCACT	XhoI	Inverse PCR
jhp0785_XhoI_inv2		atactcgagTACTGATTTACAAGCCGGTA	XhoI	
jhp0786_PstI_for	JHP0786	atactgcagATGGAAAACACAAACAAAGCGA	PstI	Cloning
jhp0786_BamHI_rev		ataggatccTCTAGCTCTTTGATGATGGA	BamHI	
jhp0786_XhoI_inv1		atactcgagGTTGGTAATCGCCTAATTGCA	XhoI	Inverse PCR
jhp0786_XhoI_inv2		atactcgagAGCATGAAGACGATGAGCCT	XhoI	
jhp1012_PstI_for	JHP1012	atactgcagATGCGCATGCCAATTATGTC	PstI	Cloning
jhp1012_BamHI_rev		ataggatccTTGGTCTTCGCACACTCCTA	BamHI	
jhp1012_BglII_inv1		ataagatctAGGTTCAATGTCTATGCCAA	BglII	Inverse PCR
jhp1012_BglII_inv2		ataagatctAGGCTCTATCAAGTATCGCGT	BglII	
jhp1271_PstI_for	JH1271	atactgcagTGTAACGGGCATGCTTTGCG	PstI	Cloning
jhp1271_BamHI_rev		ataggatccACTTAATCTCATGGGAAGTCA	BamHI	
jhp1271_BglII_inv1		ataagatctGCTCTTGTGCCAAACAATAT	BglII	Inverse PCR
jhp1271_BglII_inv2		ataagatctGCGCGACTAAACCTAAAGAC	BglII	
jhp1284_PstI_for	JHP1284	atactgcagGTGAAAAGCGAAACGATT	PstI	Cloning
jhp1284_BamHI_rev		ataggatccTTACCAAATGAGCGCTTCT	BamHI	
jhp1284_BglII_inv1		ataagatctTTCGCTCACTAATGCAATCA	BglII	Inverse PCR
jhp1284_BglII_inv2		ataagatctAGATAGCACATGGCTAAGCCT	BglII	
jhp1296_PstI_for	JHP1296	atactgcagATGTTAAAAACCCCACTCAA	PstI	Cloning
jhp1296_BamHI_rev		ataggatccCGCGTTGAATGATCAAACTT	BamHI	
jhp1296_XhoI_inv1		atactcgagGTCAATGTAGATGCATTTCA	XhoI	Inverse PCR
jhp1296_XhoI_inv2		atactcgagAAGGGAGTGATTAAGCCTAC	XhoI	

jhp1365_PstI_for	JHP1365	atactgcagGTAACCGCCCTGATGTGAAA	PstI	Cloning
jhp1365_BamHI_rev		ataggatccTCATAGTAGTCATCTTCGCT	BamHI	
jhp1365_BglII_inv1		ataagatctTGAGCTGCATTAAGCAGTCT	BglII	Inverse PCR
jhp1365_BglII_inv2		ataagatctAGCCAGACTCTCCAAAGTCA	BglII	
jhp1423_PstI_for	JHP1423	atactgcagATCGCAGAGCGAAACGACTT	PstI	Cloning
jhp1423_BamHI_rev		ataggatccTTCGCTTCTAGCCATTCTA	BamHI	
jhp1423_BglII_inv1		ataagatctGTTAGAAAGGGTGCTAGAAC	BglII	Inverse PCR
jhp1423_BglII_inv2		ataagatctTGATCGATGCGAGCAAGGAT	BglII	
Km8_for_XhoI	*aphA3* cassette	atactcgagAGCGAACCATTTGAGGTG	XhoI	Amplification of *aphA3* from pILL600
Km9_rev_XhoI		atactcgagATCATCGATAAGCTTTTTAGAC	XhoI	
Km8_for_BamHI		tatggatccAGCGAACCATTTGAGGTG	BamHI	
Km9_rev_BamHI		tatggatccATCATCGATAAGCTTTTTAGAC	BamHI	

* RS: Restriction sites.

Table S3: Oligonucleotide primers used for expression of candidate genes in *E. coli*.

Primer	Target gene	5’ → 3’ sequence	RS*	Application
26695 orf463 exp for meth	HP0462-HP0463	tgcctgcagttaaggtttaacatATGCCTAATAACGCTTTATTGCAAATCAAACAAGAC	PstI	Expression
26695 orf463 exp rev S		tctagatcttccccggggatccTTATCATTGGGGTTTGACTTGCTGTTTTAAGAGTA	BamHI	
26695 orf668 exp for	HP0667-HP0668-HP0669	tgcctgcagttaaggtttaacatATGCAAGAAATCAGTGCCTATACACTCATTAAA	PstI	Expression
26695 orf668 INTERNAL exp for		tgcctgcagttaaggtttaacatATGACCCAAGCCTGGTTGATGAAG	PstI	
26695 orf668 exp rev		tctagatcttccccggggatccTTATCACTCAAACCTCTTTTCGCTGATCTT	BamHI	
26695 orf 668 fix fs1 for		CCTTCAAACAAAGAATACATCGCTGTGCAATGCAAA	-	Frameshift correction (mut1)
26695 orf 668 fix fs1 rev		CGTAATGACTATATCAATCCCCTTATCGCGCTCATT	-	
26695 orf 668 fix fs2 for		ATGGCAACCAATCACAACGATGAAAAAACCCTT	-	Frameshift correction (mut2)
26695 orf 668 fix fs2 rev		GCTTCCAAAGATTTTGATTTTTTCTTTAAAAGTGGCTTCATC	-	
26695orf668 1880F		TAGAAGAGAGTGAAATCCAAAACCTGGATGAAG	-	Sequencing (mut2)
26695 orf 810 exp for	HP0810	tgcctgcagttaaggtttaacatATGCCAAATCATCAGCCAGTAAAAAAATTTAAGA	PstI	Expression
26695 orf 810 exp rev		tctagatcttccccggggatccTTACTATTGAAAATAAGTTAAAGTGGTTTTTCCAA	BamHI	
hp0910_XbaI_for	HP0910	atatctagaTTGAATAATTTAGACATTAAAACT	-	Expression
hp0910_XbaI_rev		atatctagaTTATCCTAGATTTAAAAAGTCT	-	
hp0910_1s		ACGCAATTAGGCATGGCGTT	-	Sequencing
hp0910_2s		TTGAACTGGGCGATCAAA	-	
HP0910METH exp for (long gene)		tgcctgcagttaaggtttaacatATGGAGAATTTTTTGAATAATTTAGACATTAAAACTT	PstI	Expression
HP0910METH exp rev (long gene)		tctagatcttccccggggatccTTATCCTAGATTTAAAAAGTCTTTAGGCAAAAG	BamHI	
HP0909 ENDO exp for		tgcctgcagttaaggtttaacatATGATACCCACACAGCTTAATGAAATTGCAGAA	PstI	
26695 orf1353 exp for	HP1353-HP1354	tgcctgcagttaaggtttaacatATGCTAAAAGAATATTTAGAAAGCATTAAAGATCTT	PstI	Expression
26695 orf1353 exp rev		tctagatcttccccggggatccTTACTATTTTAAACGATACAACAAAAACGCATAACT	BamHI	
26695 orf1353 fix fs1 for		CCTCCAACAAACCCTAAAACACCCAATCAAACG	-	Frameshift correction

26695 orf1353 fix fs1 rev		CGGAGGTAACATGTGCTTAAAAACTTCGCCTCGT	-	(mut1)
26695 orf1353 fix fs2 for		CCTCCCCATTTAATACCAATATCGCCCTTATCTTAT	-	Frameshift correction (mut2)
26695 orf1353 fix fs2 rev	HP1353-HP1354	TGGGGGTGGATTTTGCAAGATTTCTTGCAT	-	
26695orf1353 2347F		AAGGCAAACGCTAACAATTTAGAAGAATACATTGTT	-	Sequencing (mut1)
26695orf1353 3180F		TATCATTAAAGTCATCGCAACGCACGATTGA	-	Sequencing (mut2)
26695 orf1370 exp for	HP1369-HP1370	tgcctgcagttaaggtttaacatATGAAAACGAACGAAGCGCAATTTTATGAA	PstI	Expression
26695 orf1370 exp rev		tctagatcttccccggggatccTTACCAAATGAGCGCTTCTTTTAAGGCTTT	BamHI	
26695 orf1370 fix fs for		GATAACTTTGTAGCGACTATTAGTTGGAAACAATTTCAT	-	Frameshift correction
26695 orf1370 fix fs rev		CACGCCACCCCCATTAAACACTTCGTCCATGAG	-	
26695orf1370 1280F		CTATGATGGAAAACCGCTTGGAGCTTG	-	Sequencing
hp1471-72_PstI_new_for	HP1471-HP1472	tgcctgcagttaaggtttaacatATGAATAAAGTTCAATCTATTG	PstI	Expression
hp1471-72_BamHI_rev		ataggatccTTAAAATAACGAGTCTTTTTGA	BamHI	
hp1471-72_fix_joint_for		TAATATATGGGCCCCCTTAATAGCCAACTC	-	Correction of joint between hp1472 & hp1471
hp1471-72_fix_joint_rev		ACCTTATCGCTTTGGGGGCATTTGGTT	-	
hp1471-72_fix_fs_for		GGAGGTAATACCCCATGCGGCTTAA	-	Frameshift correction
hp1471-72_fix_fs_rev		TCCACCACCAGAATTATTGAAAACA	-	
26695 orf1522 exp for	HP1522	tgcctgcagttaaggtttaacatATGCAAAATAAAGAAATTGGTGAAGAAAAAAGCGTT	PstI	Expression
26695 orf1522 exp rev		tctagatcttccccggggatccTTACTACCCCCTAATCTTTAAATCGCCACTCTC	BamHI	
26695 orf1522 fix fs for		GGAGGCTTATTTAATGGTTTGAACGCCGCATTT	-	Frameshift correction
26695 orf1522 fix fs rev		GCCACCCTCACTTAATTTTTGATAATCGCTCTT	-	
26695orf1522 1472R		TTGGGGAGGGTCATTTTAAGGTGTTCAAAC	-	Sequencing
J99 orf 0415 exp for	JHP0414-JHP0415	tgcctgcagttaaggtttaacatATGCCTAATAACGCTTTATTGCAAATCAAACA	PstI	Expression
J99 orf 0414 exp rev		tctagatcttccccggggatccTTATCATTGTGGTTTGACTTGTTGTTTTAAGAGTAG	BamHI	
J99 orf 0414 175 rev		ATCGTGAAATGCTGCGGCTGC	-	Sequencing
J99 orf 0415 638 for		TAAGCATCGCTAGCATCATTGCCA	-	
J99 orf 0414 exp for		tgcctgcagttaaggtttaacatATGAGTGAGTGGCAAACATT	PstI	

		TTGTTT		
J99 orf 0415 exp rev		tctagatcttccccggggatccTTATCACTCAAACCTAACCCCTTTTAAAGTTTCT	BamHI	
J99 orf612 meth FOR	JHP0612	tgcctgcagttaaggtttaacatATGCAAGAAATCAGTGCCTACGAACTCATC	PstI	Expression
J99 orf612 meth REV		tctagatcttccccggggatccTTATCACTCAAACCTCTTCTCGCTGATCTTTTCA	BamHI	
J99 orf612 Fix FS FOR		GGCACTAAATTGGATAAAAAGCTTATTAATAACGAA	-	Frameshift correction
J99 orf612 Fix FSREV		TTTGGCTTCAAGCTGGCTGATTTTCTT	-	
J99orf612 1220F		TTGAAAGAGCGATCGCTTTAGATCTCTTAA	-	Sequencing
J99 orf 0726 exp for	JHP0726	tgcctgcagttaaagtttaacatATGAACAAAATAGAGTTATTACTCCACACTCTAGC	PstI	Expression
J99 orf 0726 exp rev		tctagatcttccccggggatccTTATCATTTTTTAACTTCTTTATTGAGGGTTAGGGGT	BamHI	
J99 orf 0786 exp for	JHP0785 - JHP0786	tgcctgcagttaaagtttaacatATGGAAAACACAAACAAAGCGAGCG	PstI	Expression
J99 orf 0785 exp rev		tctagatcttccccggggatccTTATTACGCAAGCTCTTTGTTATTTAGTGGG	BamHI	
J99 orf 0785 exp for		tgcctgcagttaaagtttaacatATGAATAAAATAGAGCGATTACTCCAAACTCTAGC	PstI	Sequencing
J99 orf 0786 exp rev		tctagatcttccccggggatccTTATTAAATTTCTAGCTCTTTGATGATGGATTCAAGG	BamHI	
J99 orf 0786 1455 for		CTACGTGGAGCAAGAAGACACGA	-	
J99 orf1272 meth FOR	JHP1272	tgcctgcagttaaggtttaacatATGCTAAAAGAATATTTAGAAAGCATTAAAGAT	PstI	Expression
J99 orf1272 meth REV		tctagatcttccccggggatccTTACTATTTTAATCGGTATAATAAAAACGCATAACTC	BamHI	
J99 orf1272 Fix FS1 FOR		CCACCGCCAACAAACCCTAAAACACCCAATCAAACG	-	Frameshift correction (wt)
J99 orf1272 Fix FS1 REV		TGGGGGTAACATGTGCTTGAATGTGTTCTC	-	
J99 orf1272 Fix FS2 FOR		GGGTGGATTTTGCAAGATTTCTTGTATTAAGGCTTT	-	Frameshift correction (mut1)
J99 orf1272 Fix FS2 REV		CCTCCACCCCATTTAATGCCAATATCGCCCTTATCT	-	
J99 orf 1272 610 for		AGTTTTGAAGACTTTAGCGATGCGT	-	Sequencing
J99 orf 1272 1276 for		AAGCCTCTCAAAGAAAACGACAC	-	
J99 orf 1272 1806 for		AAGGCGCTCTCTTTTAGAATGCTA	-	
J99orf1272 2520R		CCCAGAGCTTAACCCTTGATCGTTAAGTTG	-	Sequencing (wt)
J99orf1272 3350R		TAAAGGGAAGGCTGAACCTCCG	-	Sequencing (mut1)
J99 orf1284 meth FOR	JHP1284	tgcctgcagttaaggtttaacatATGAAAAGCGAAACGATTTATAAGGATTTTTGCTT	PstI	Expression

J99 orf1284 meth REV		tctagatcttccccggggatccTTACCAAATGAGCGCTTCTTTTAAGGCTTT	BamHI	
jhp1284 endo rev	JHP1285	tctagatcttccccggggatccTTATCAGGCTAATCCTTTAAATCCAGAAAA	BamHI	Expression
J99 orf1296 meth FOR	JHP1296	tgcctgcagttaaggtttaacatATGTTAAAAACCCCACTCAAAACCCTA	PstI	Expression
J99 orf1296 meth REV		tctagatcttccccggggatccTTAAGAATAAAAAATTTCATTGTATTTTTTGGTGATCT	BamHI	
jhp1296 endo for	JHP1297	tgcctgcagttaaggtttaacatATGTTTGAAATGGCGACCGGTAGC	PstI	Expression
jhp1364-65_PstI_for	JHP1364 - JHP1365	tgcctgcagttaaggtttaacatATGAATAAAGTTCAATCTATTGAG	PstI	Expression
jhp1364-65_BamHI_rev		ataggatccTCATCTATTTCAAAATAACGAGT	BamHI	
jhp1364-65_fix_joint_for		TAATATATGGGCCCCCTTAGCAGCCAACT	-	Correction of joint between jhp1365 & jhp1364
jhp1364-65_fix_joint_rev		ACCTTATCACTTTGGGGGCGTTTGGTTTT	-	
hp1471-72_fix_fs_for		GGAGGTAATACCCCATGCGGCTTAA	-	Frameshift correction of JHP1364
hp1471-72_fix_fs_rev		TCCACCACCAGAATTATTGAAAACA	-	
J99orf1409 meth for	JHP1409	AAACCTGCAGGTAAGGTGGACATATGAGTTTCACTCGCATCCCCCTTAAAGTTTTC	SbfI	Expression
J99orf1409 meth rev		AAAGGATCCTTACTGCCCGTCTTCAATGGTTTTAATTTCTTCATCG	BamHI	
J99 orf1411 meth FOR	JHP1411	tgcctgcagttaaggtttaacatATGCAAAATAAAGAAATTGGTGGAGAAAAAAGCGT	PstI	Expression
J99 orf1411 meth REV		tctagatcttccccggggatccTTACTACCCCCTAATCTTTAAATCGCCGCTCT	BamHI	
J99 orf1411 Fix FS FOR		GGTGGGTTATTTAATGGCTTGAACGCCGTATTT	-	Frameshift correction
J99 orf1411 Fix FS REV		GCCACCCTCACTTAATTTTTGATAATCGCTCTTATTACTCTC	-	
J99orf1411 1460R		CTCGGCGAGGTGGATTTTAAGGTGTTC	-	Sequencing
J99 orf 1423 exp for	JHP1422 - JHP1423	tgcctgcagttaaagtttaacatATGGCGATCAAAAAAAGCGAATTGTATAGC	PstI	Expression
J99 orf 1422 exp rev		tctagatcttccccggggatccTTATTATTTGTTTAAAACCCTGATTTTAGCGCTC	BamHI	
J99 orf 1422 exp for		tgcctgcagttaaagtttaacatATGATGGAACGCATGGACGCTTT	PstI	Sequencing
J99 orf 1423 exp rev		tctagatcttccccggggatccTTATCACCCCATTAACCCCAAATCTTTCA	BamHI	
jhp1423_Pos600_for		CGGTTCGTTATTGTTAAAAGC	-	
jhp1423_Pos1478_for		AGTCTTGCAAGACGATTGGTTC	-	

jhp1423_Pos1813_rev		TCCTCAATTCTTTAGCGCTT	-	
jhp1423_Pos2300_for		AGATCCAAAAACGCACCAACG	-	
jhp1422_Pos574_for		ATTCTTAAAAAAGAAAGCGTT	-	
J99 orf 1422 TRDdel1 for_new	JHP1422	TGAGCGCTAAAATCAGGGTTTTAAACA	-	Generation of TRD deletion mutants of JHP1422
J99 orf 1422 TRDdel2 for		ATGCGCCAATTTGAAAATGTCAAAAAAGCCTTA	-	
J99 orf 1422 TRDdel3 for		ATGCTTAAAAAAGAAAGCGTTAAAAAAGCTTTAAGCTTT	-	
J99 orf 1422 TRDdel4 rev		AAGGCTTTTTTGACATTTTCAAATTGGCGTT	-	
J99 orf 1422 TRDdel5 rev		GCTTAAAGCTTTTTTAACGCTTTCTTTTTTAAGAATGAG	-	
J99 orf 1422 TRDdel6 rev		CACCCCATTAACCCCAAATCTTTCAAATGCTCT	-	
jhp1422_6_fs_for		GGCGATCAAAAAAAGCGAATTGTATAGC	-	Correction of SNP in allele variant 4
jhp1422_6_fs_rev		ATATGTTAAACCTTAACTGCAGGCAT	-	
S1224P	-	CGCCAGGGTTTTCCCAGTCACGAC	-	Sequencing of pRRS inserts
S1233P		AGCGGATAACAATTTCACACAGGA	-	
NEB1219	-	ATGCGTCCGGCGTAGA	-	Sequencing pACYC184 inserts
NEB1245		AGAATTCTCATGTTTGACAGCTTATCATCG	-	
pACYC184 GIBSON for	-	TAAGGATCCCCGGGGAAGATCTAGAGGCTTGGTTATGCCGGTACTGCC	-	Amplification of pACYC184 for Gibson cloning
pACYC184 GIBSON "A" rev		CATATGTTAAACTTTAACTGCAGGCAGCATCCAGGGTGACGGTGCCGA	-	
pRRS srbs	-	CATATGTTAAACCTTAACTGCAGGCATGCAAGCTTGGC	-	Amplification of pRRS for Gibson cloning
pRRS rev		TAAGGATCCCCGGGGAAGATCTAGA	-	

* RS: Restriction sites.

Table S4: Plasmids used in this study.

Plasmids	Genotype	Source
pACYC184	Cm^r, Tc^r, p15A Ori	(7,8)
pILL600	Source of the *aphA-3* cassette	(9)
pRRS	pUC19 derivative, retroregulator structure from *B. thuringiensis* inserted into the BglII linker of pBBO (pUC19 modified by insertion of a BglII linker at the SacI site)	(10)
pRRS_HpyAIXP	Amp^r, pRRS derivative containing *hp0909-hp0910*	This study
pRRS_M.HpyAIXP	Amp^r, pRRS derivative containing *hp0910*	This study
pRRS_jhp1409	Amp^r, pRRS derivative containing *jhp1409*	This study
pSUS2632	Amp^r, Km^r, *H. pylori ureAB* fragment in pUC19 with *aphA-3* cassette	(12)
pSUS3016	Amp^r, pUC19 derivative containing *jhp0085*	This study
pSUS3017	Amp^r, relegated inverse PCR of pSUS3016	This study
pSUS3018	Amp^r, Km^r, pSUS3017 derivative with a *jhp0085::aphA-3* disruption	This study
pSUS3019	Amp^r, pUC19 derivative containing *jhp1271*	This study
pSUS3020	Amp^r, relegated inverse PCR of pSUS3019	This study
pSUS3021	Amp^r, Km^r, pSUS3020 derivative with a *jhp1271::aphA-3* disruption	This study
pSUS3024	Amp^r, pUC19 derivative containing *jhp0430*	This study
pSUS3025	Amp^r, pUC19 derivative containing *jhp1012*	This study
pSUS3026	Amp^r, relegated inverse PCR of pSUS3024	This study
pSUS3027	Amp^r, relegated inverse PCR of pSUS3025	This study
pSUS3028	Amp^r, pUC19 derivative containing *hp0850*	This study
pSUS3029	Amp^r, pUC19 derivative containing *hp1369*	This study
pSUS3030	Amp^r, pUC19 derivative containing *hp1370*	This study
pSUS3031	Amp^r, pUC19 derivative containing *hp1403*	This study
pSUS3032	Amp^r, pUC19 derivative containing *hp1472*	This study
pSUS3033	Amp^r, pUC19 derivative containing *hp1517*	This study
pSUS3035	Amp^r, pUC19 derivative containing *jhp0612*	This study
pSUS3036	Amp^r, pUC19 derivative containing *jhp0786*	This study
pSUS3037	Amp^r, pUC19 derivative containing *jhp1284*	This study
pSUS3038	Amp^r, pUC19 derivative containing *jhp1296*	This study
pSUS3039	Amp^r, pUC19 derivative containing *jhp1365*	This study
pSUS3040	Amp^r, pUC19 derivative containing *jhp1423*	This study
pSUS3041	Amp^r, Km^r, pSUS3025 derivative with a *jhp1012::aphA-3* disruption	This study
pSUS3042	Amp^r, Km^r, pSUS3026 derivative with a *jhp0430::aphA-3* disruption	This study
pSUS3043	Amp^r, relegated inverse PCR of pSUS3028	This study
pSUS3044	Amp^r, relegated inverse PCR of pSUS3029	This study
pSUS3045	Amp^r, relegated inverse PCR of pSUS3030	This study

pSUS3046	Amp^r, relegated inverse PCR of pSUS3031	This study
pSUS3047	Amp^r, relegated inverse PCR of pSUS3032	This study
pSUS3048	Amp^r, relegated inverse PCR of pSUS3033	This study
pSUS3050	Amp^r, relegated inverse PCR of pSUS3035	This study
pSUS3051	Amp^r, relegated inverse PCR of pSUS3036	This study
pSUS3052	Amp^r, relegated inverse PCR of pSUS3037	This study
pSUS3053	Amp^r, relegated inverse PCR of pSUS3038	This study
pSUS3054	Amp^r, relegated inverse PCR of pSUS3039	This study
pSUS3055	Amp^r, relegated inverse PCR of pSUS3040	This study
pSUS3056	Amp^r, Km^r, pSUS3043 derivative with a *hp0850::aphA-3* disruption	This study
pSUS3057	Amp^r, Km^r, pSUS3044 derivative with a *hp1369::aphA-3* disruption	This study
pSUS3058	Amp^r, Km^r, pSUS3045 derivative with a *hp1370::aphA-3* disruption	This study
pSUS3059	Amp^r, Km^r, pSUS3046 derivative with a *hp1403::aphA-3* disruption	This study
pSUS3061	Amp^r, Km^r, pSUS3048 derivative with a *hp1517::aphA-3* disruption	This study
pSUS3063	Amp^r, Km^r, pSUS3050 derivative with a *jhp0612::aphA-3* disruption	This study
pSUS3064	Amp^r, Km^r, pSUS3051 derivative with a *jhp0786::aphA-3* disruption	This study
pSUS3065	Amp^r, Km^r, pSUS3052 derivative with a *jhp1284::aphA-3* disruption	This study
pSUS3067	Amp^r, Km^r, pSUS3054 derivative with a *jhp1365::aphA-3* disruption	This study
pSUS3068	Amp^r, Km^r, pSUS3055 derivative with a *jhp1423::aphA-3* disruption	This study
pSUS3069	Amp^r, Km^r, pSUS3047 derivative with a *hp1472::aphA-3* disruption	This study
pSUS3070	Amp^r, Km^r, pSUS3053 derivative with a *jhp1296::aphA-3* disruption	This study
pSUS3071	Amp^r, Km^r, pSUS2632 derivative with *hp0910*	This study
pSUS3072	Amp^r, pUC19 derivative containing *jhp0726*	This study
pSUS3074	Amp^r, pUC19 derivative containing *jhp0414*	This study
pSUS3075	Amp^r, pUC19 derivative containing *hp0462*	This study
pSUS3076	Amp^r, Km^r, pSUS3072 derivative with a *jhp0726::aphA-3* disruption	This study
pSUS3078	Amp^r, Km^r, pSUS3074 derivative with a *jhp0414::aphA-3* disruption	This study
pSUS3079	Amp^r, Km^r, pSUS3075 derivative with a *hp0462::aphA-3* disruption	This study
pSUS3080	Amp^r, pUC19 derivative containing *hp1522*	This study
pSUS3081	Amp^r, Km^r, pSUS3080 derivative with a *hp1522::aphA-3* disruption	This study
pSUS3082	Amp^r, pRRS derivative containing *hp1472-hp1471*	This study
pSUS3083	Amp^r, pRRS derivative containing *jhp1365-jhp1364*	This study
pSUS3084	Amp^r, pSUS3082 with joint between RM and S corrected	This study
pSUS3085	Amp^r, pSUS3083 with joint between RM and S corrected	This study
pSUS3086	Amp^r, pSUS3084 with frameshift corrected	This study
pSUS3087	Amp^r, pSUS3085 with frameshift corrected	This study
pSUS3088	Amp^r, pRRS derivative containing *jhp1422-jhp1423*, allele variant 1 (TRD1-TRD2-TRD3)	This study
pSUS3089	Amp^r, pRRS derivative containing *jhp1422-jhp1423*, allele variant 2 (TRD1-TRD2-TRD2-TRD3)	This study
pSUS3090	Amp^r, pRRS derivative containing *jhp1422-jhp1423* (TRD1-TRD3)	This study

pSUS3091	Amp^r, pRRS derivative containing *jhp1272*	This study
pSUS3092	Amp^r, pSUS3088 with frameshift 1 corrected (wt)	This study
pSUS3093	Amp^r, pSUS3089 with both frameshifts corrected (-mut1)	This study
pSUS3094	Amp^r, pRRS derivative containing *hp1353-hp1354*	This study
pSUS3095	Amp^r, pSUS3094 with frameshift 1 corrected (-mut1)	This study
pSUS3096	Amp^r, pSUS3095 both frameshifts corrected (-mut2)	This study
pSUS3097	Amp^r, pRRS derivative containing *jhp0414-jhp0415*	This study
pSUS3098	Amp^r, pRRS derivative containing *jhp0785-jhp0786*	This study
pSUS3099	Amp^r, pRRS derivative containing *jhp0786*	This study
pSUS3100	Amp^r, pACYC184 derivative containing *jhp0726*	This study
pSUS3101	Amp^r, pRRS derivative containing *hp1369-hp1370*	This study
pSUS3102	Amp^r, pSUS3101 with frameshift corrected	This study
pSUS3103	Amp^r, pRRS derivative containing *hp1522*	This study
pSUS3104	Amp^r, pSUS3103 with frameshift corrected	This study
pSUS3105	Amp^r, pRRS derivative containing *jhp1411*	This study
pSUS3106	Amp^r, pSUS3105 with frameshift corrected	This study
pSUS3107	Amp^r, pRRS derivative containing *hp0667-hp0668-hp0669*	This study
pSUS3108	Amp^r, pSUS3107 with frameshift 1 corrected	This study
pSUS3109	Amp^r, pSUS3108 with both frameshifts corrected	This study
pSUS3110	Amp^r, pRRS derivative containing *jhp0612-jhp0613*	This study
pSUS3111	Amp^r, pSUS3110 with frameshift corrected	This study
pSUS3112	Amp^r, pRRS derivative containing *hp0810*	This study
pSUS3113	Amp^r, pUC19 derivative containing *jhp0785*	This study
pSUS3114	Amp^r, Km^r, pSUS3113 derivative with a *jhp0785::aphA-3* disruption	This study
pSUS3119	Amp^r, pUC19 derivative containing *hp0790*	This study
pSUS3120	Amp^r, Km^r, pSUS3119 derivative with a *hp0790::aphA-3* disruption	This study
pSUS3121	Amp^r, pUC19 derivative containing *hp0848-49*	This study
pSUS3122	Amp^r, Km^r, pSUS3121 derivative with a *hp0848-89::aphA-3* disruption	This study
pSUS3123	Amp^r, pSUS3088 derivative containing deletion of TRD2 & 3, allele variant 4 (TRD1 only)	This study
pSUS3124	Amp^r, pSUS3089 derivative containing deletion of TRD1 & 2, allele variant 5 (TRD2 & TRD3 only)	This study
pSUS3125	Amp^r, pSUS3090 derivative containing optimised RBS, allele variant 3 (TRD1 & 3)	This study
pSUS3126	Amp^r, pSUS3090 derivative containing optimised RBS and deletion of TRD3, allele variant 4 (TRD1 only)	This study
pSUS3127	Amp^r, pSUS3090 derivative containing optimised RBS and deletion of TRD1, allele variant 6 (TRD3 only)	This study
pSUS3128	Amp^r, pRRS derivative containing *jhp1422-jhp1423* (TRD1)	This study
pSUS3129	Amp^r, pSUS3128 derivative with SNP repaired in *jhp1423* gene,	This study

	allele variant 4 (TRD1)	
pUC19	Amp^r, Colx101, MCS within *lacZ*: blue/white selection	(11)

Table S5: Comparison of detected recognition sites in *H. pylori* 26695 and isogenic MTase or specificity subunit mutant strains.

**26695 wt[a,b]**	**26695hp0462**	**26695hp0790**	**26695hp00848-49**	**26695hp0850**	**26695hp1369**	**26695hp1370**	**26695hp1403**	**26695hp1472**	**26695hp1517**	**26695hp1522**
ACAN$_8$TAG*	ACAN$_8$TAG	-	ACAN$_8$TAG	-	ACAN$_8$TAG	ACAN$_8$TAG	ACAN$_8$TAG	ACAN$_8$TAG	ACAN$_8$TAG	ACAN$_8$TAG
ATTAAT	ATTAAT	ATTAAT	ATTAAT	ATTAAT	ATTAAT	ATTAAT	ATTAAT	ATTAAT	ATTAAT	ATTAAT
CATG	CATG	CATG	CATG	CATG	CATG	CATG	CATG	CATG	CATG	CATG
CGRAT*	CGRAT	CGRAT	CGRAT	CGRAT	CGRAT	CGRAT	CGRAT	CGRAT	CGRAT	CGRAT
GAAGA	GAAGA	GAAGA	GAAGA	GAAGA	GAAGA	GAAGA	GAAGA	GAAGA	GAAGA	GAAGA
GAAGG[b]	GAAGG	GAAGG	GAAGG	GAAGG	GAAGG	GAAGG	GAAGG	GAAGG	GAAGG	GAAGG
GAGG[b]	GAGG	GAGG	GAGG	GAGG	GAGG	GAGG	GAGG	GAGG	GAGG	GAGG
GANTC	GANTC	GANTC	GANTC	GANTC	GANTC	GANTC	GANTC	GANTC	GANTC	GANTC
GATC	GATC	GATC	GATC	GATC	GATC	GATC	GATC	GATC	GATC	GATC
GCAG	GCAG	GCAG	GCAG	GCAG	GCAG	GCAG	GCAG	GCAG	GCAG	GCAG
GCGTA*	GCGTA	GCGTA	GCGTA	GCGTA	GCGTA	GCGTA	GCGTA	GCGTA	-	GCGTA
TCGA	TCGA	TCGA	TCGA	TCGA	TCGA	TCGA	TCGA	TCGA	TCGA	TCGA

[a] Novel recognition sites are labelled with an asterisk.

[b] The table only contains recognition sites methylated by ^{m6}A and ^{m4}C MTases because detection of ^{m5}C methylation (requires TET-treatment) was not performed for the low coverage SMRT sequencing carried out for the mutant strain analyses. In the case of the ^{m5}C recognition sites CCTTC and CCTC, only the reverse complementary sequences GAAGG and GAGG, respectively, were detected (^{m6}A methylation) and presented in the table.

Table S6: Comparison of detected recognition sequences in *H. pylori* J99-R3 and isogenic MTase or specificity subunit mutant strains.

**J99-R3 wt**[a,b]	**J99-R3 jhp0414**	**J99-R3 jhp0612**	**J99-R3 jhp0726**[c]	**J99-R3 jhp0785**	**J99-R3 jhp0786**	**J99-R3 jhp1284**	**J99-R3 jhp1296**	**J99-R3 jhp1365**	**J99-R3 jhp1423**
AAGN$_5$CTT*	AAGN$_5$CTT	AAGN$_5$CTT	AAGN$_5$CTT	AAGN$_5$CTT	AAGN$_5$CTT	AAGN$_5$CTT	AAGN$_5$CTT	AAGN$_5$CTT	-
AAGN$_6$CTC*	AAGN$_6$CTC	AAGN$_6$CTC	-	AAGN$_6$CTC	-	AAGN$_6$CTC	AAGN$_6$CTC	AAGN$_6$CTC	AAGN$_6$CTC
AAGN$_6$TAAAG*	AAGN$_6$TAAAG	AAGN$_6$TAAAG	AAGN$_6$TAAAG	AAGN$_6$TAAAG	AAGN$_6$TAAAG	AAGN$_6$TAAAG	AAGN$_6$TAAAG	AAGN$_6$TAAAG	-
ATTAAT	ATTAAT	ATTAAT	ATTAAT	ATTAAT	ATTAAT	ATTAAT	ATTAAT	ATTAAT	ATTAAT
CATG	CATG	CATG	CATG	CATG	CATG	CATG	CATG	CATG	CATG
CCGG	CCGG	CCGG	CCGG	CCGG	CCGG	CCGG	CCGG	CCGG	CCGG
CCNNGG	CCNNGG	CCNNGG	CCNNGG	CCNNGG	CCNNGG	CCNNGG	CCNNGG	CCNNGG	CCNNGG
CGWCG	CGWCG	CGWCG	CGWCG	CGWCG	CGWCGC	CGWCG	CGWCG	CGWCG	CGWCG
GAGG[b]	GAGG	GAGG	GAGG	GAGG	GAGG	GAGG	GAGG	GAGG	GAGG
GANTC	GANTC	GANTC	GANTC	GANTC	GANTC	GANTC	GANTC	GANTC	GANTC
GATC	GATC	GATC	GATC	GATC	GATC	GATC	GATC	GATC	GATC
GCCTA*	GCCTA	GCCTA	GCCTA	GCCTA	GCCTA	GCCTA	GCCTA	GCCTA	GCCTA
GGWTAA*	GGWTAA	GGWTAA	GGWCNA[c]	GGWTAA	GGWTAA	GGWTAA	GGWTAA	GGWTAA	GGWTAA
GTAC	GTAC	GTAC	GTAC	GTAC	GTAC	GTAC	GTAC	GTAC	GTAC
GTSAC	GTSAC	GTSAC	GTSAC	GTSAC	GTSAC	GTSAC	GTSAC	GTSAC	GTSAC
RTAYN$_5$RTAY*	RTAYN$_5$RTAY	RTAYN$_5$RTAY	RTAYN$_5$RTAY	-	-	RTAYN$_5$RTAY	RTAYN$_5$RTAY	RTAYN$_5$RTAY	RTAYN$_5$RTAY
TCGA	TCGA	TCGA	TCGA	TCGA	TCGA	TCGA	TCGA	TCGA	TCGA
TCNNGA	TCNNGA	TCNNGA	TCNNGA	TCNNGA	TCNNGA	TCNNGA	TCNNGA	TCNNGA	TCNNGA

[a] Novel recognition sites are labelled with an asterisk.

[b] The table only contains recognition sites methylated by ^{m6}A and ^{m4}C MTases because detection of ^{m5}C methylation (requires TET-treatment) was not performed for the low coverage SMRT sequencing carried out for the mutant strain analyses. In the case of the ^{m5}C recognition site CCTC, only the reverse complementary sequence GAGG was detected (^{m6}A methylation) and presented in the table.

[c] In our study, the J99-R3 MTase JHP1272 was found to catalyse modification of either GGWTAA (first repeat in frame, Hpy99XIV) or GGWCNA (full length protein, Hpy99XIV-mut1) depending on the length of its C-terminus. Except for the JHP0726 mutant, which contains Hpy99XIV-mut1; strain J99-R3 and all mutants encode Hpy99XIV wt.

Table S7: Methylation status of identified motifs (^{m6}A and ^{m4}C)

Motif	No. detected as methylated	No. detected as non-methylated[a]	No. uncalled[b]	No. detected methylated QV40 < score < QV100[c]	Theoretical no. of motifs present in the genomes
	***H. pylori* 26695**				
ACAN$_8$TAG	322	5	0	3	330
ATTAAT	937	23	0	12	972
CATG	14662	80	31	9	14782
CGRAT	3160	92	12	12	3276
CTAN$_8$TGT	322	5	1	2	330
GAAGA	4747	65	7	4	4823
GAAGG	1240	21	7	37	1305
GAGG	4730	118	25	14	4887
GANTC	5512	40	17	3	5572
GATC	10647	123	57	7	10834
GCAG	4207	33	18	3	4261
GCGTA	2026	22	8	2	2058
TCGA	589	14	4	1	608
TCTTC	3958	690	22	153	4823
	***H. pylori* J99-R3**				
AAGN$_5$CTT	1561	5	4	12	1582
AAGN$_6$CTC	610	0	1	0	611
AAGN$_6$TAAAG	283	2	0	2	287
ATTAAT	822	11	1	20	854
CATG	15039	14	10	57	15120
CCGG	3044	24	7	549	3624
CCNNGG	1780	47	13	558	2398
CGWCG	217	51	3	267	538
CTTTAN$_6$CTT	284	1	1	1	287
GAGG	4907	19	13	88	5027
GAGN$_6$CTT	608	0	1	2	611
GANTC	5463	12	16	25	5516
GATC	10826	10	10	112	10958
GCCTA	3072	4	2	28	3106
GGWTAA	2611	12	8	45	2676
GTAC	343	3	9	13	368
GTSAC	203	2	2	3	210
RTAYN$_5$RTAY	366	0	0	4	370
TCGA	639	14	7	14	674
TCNNGA	3693	46	4	163	3906

[a] not detected is defined as coverage > 25 and low score <QV 40

[b] no call is low coverage <=25x

[c] remainder is coverage > 25 but QV is between 40 and 100, where the sites are likely partially methylated

Reference List

1. Hanahan,D. (1983) Studies on transformation of *Escherichia coli* with plasmids. *J.Mol.Biol.*, **166**, 557-580.

2. Sibley,M.H. and Raleigh,E.A. (2004) Cassette-like variation of restriction enzyme genes in *Escherichia coli* C and relatives. *Nucleic Acids Res.*, **32**, 522-534.

3. Kong,H., Lin,L.F., Porter,N., Stickel,S., Byrd,D., Posfai,J. and Roberts,R.J. (2000) Functional analysis of putative restriction-modification system genes in the *Helicobacter pylori* J99 genome. *Nucleic Acids Res.*, **28**, 3216-3223.

4. Casadaban,M.J. and Cohen,S.N. (1980) Analysis of gene control signals by DNA fusion and cloning in *Escherichia coli*. *J.Mol.Biol.*, **138**, 179-207.

5. Tomb,J.F., White,O., Kerlavage,A.R., Clayton,R.A., Sutton,G.G., Fleischmann,R.D., Ketchum,K.A., Klenk,H.P., Gill,S., Dougherty,B.A. *et al.* (1997) The complete genome sequence of the gastric pathogen *Helicobacter pylori*. *Nature*, **388**, 539-547.

6. Kulick,S., Moccia,C., Didelot,X., Falush,D., Kraft,C. and Suerbaum,S. (2008) Mosaic DNA imports with interspersions of recipient sequence after natural transformation of *Helicobacter pylori*. *PLoS.One.*, **3**, e3797.

7. Chang,A.C. and Cohen,S.N. (1978) Construction and characterization of amplifiable multicopy DNA cloning vehicles derived from the P15A cryptic miniplasmid. *J.Bacteriol.*, **134**, 1141-1156.

8. Rose,R.E. (1988) The nucleotide sequence of pACYC184. *Nucleic Acids Res.*, **16**, 355.

9. Labigne-Roussel,A., Courcoux,P. and Tompkins,L. (1988) Gene disruption and replacement as a feasible approach for mutagenesis of *Campylobacter jejuni*. *J.Bacteriol.*, **170**, 1704-1708.

10. Skoglund,C.M., Smith,H.O. and Chandrasegaran,S. (1990) Construction of an efficient overproducer clone of HinfI restriction endonuclease using the polymerase chain reaction. *Gene*, **88**, 1-5.

11. Yanisch-Perron,C., Vieira,J. and Messing,J. (1985) Improved M13 phage cloning vectors and host strains: nucleotide sequences of the M13mp18 and pUC19 vectors. *Gene*, **33**, 103-119.

12. Moccia,C., Krebes,J., Kulick,S., Didelot,X., Kraft,C., Bahlawane,C. and Suerbaum,S. (2012) The nucleotide excision repair (NER) system of *Helicobacter pylori*: role in mutation prevention and chromosomal import patterns after natural transformation. *BMC.Microbiol.*, **12**, 67.
